# Prediction of factors influencing adults‘ likelihood of accepting any COVID-19 vaccination and their willingness to pay – a nation-wide discrete choice experiment among the general public from India

**DOI:** 10.1186/s12889-025-25658-w

**Published:** 2025-12-30

**Authors:** Jeffrey Pradeep Raj, Thenmozhi Mani, Melvin Joy, Sukant Pandit, Charuta Godbole, Shital Bendkhale, Dhruve Soni, Jeyaseelan Lakshmanan, Nithya Jaideep Gogtay

**Affiliations:** 1 Division of Clinical Pharmacology, Department of Pharmacology, Kasturba Medical College, Manipal Academy of Higher Education (MAHE), Manipal, PIN - 576104 Karnataka India; 2 Department of Biostatistics, Christian Medical College, Vellore, India; 3 Department of Clinical Pharmacology, Seth GS Medical College & KEM Hospital, Mumbai, India

**Keywords:** Pharmacoeconomics, Vaccine hesitancy, Vaccine compliance, Low- and middle- income country, Informed choices

## Abstract

**Background:**

Vaccines remain a key prevention strategy against any contagion and mitigate an epidemic/pandemic. However, during the COVID-19 pandemic vaccine uptake was not as brisk as expected right from the roll out because of concerns regarding certain vaccine attributes like effectiveness, risk of adverse events, and cost among other factors that affected decision making. Thus, a discrete choice experiment (DCE) was conducted to identify the key attributes of COVID-19 vaccine for its acceptability and people’s willingness to pay for it.

**Methods:**

A pan-India digital cross-sectional survey was conducted using a non-probability convenience sampling approach among adults aged ≥ 18 years, of any gender, and residing in India for at least six months. Data were collected through the SurveyMonkey™ digital platform and distributed via organisational mailing lists and social media networks. The DCE section included six hypothetical vaccine pairs, one of which served as a trap question where one vaccine was unambiguously better than the other; respondents who failed the trap question were excluded.

**Results:**

Of 10,000 respondents, 1,241 failed the trap question, and 8,759 valid responses were included in the analysis. Vaccine effectiveness and duration of protection emerged as the key drivers of vaccine choice, with a relative attribute importance values of 54.2% and 20.6%, respectively. Respondents were willing to pay approximately INR 1,549 (USD 20.12) for a vaccine offering 90% protection (compared to 50%) and INR 587 (USD 7.62) for a 5-year duration of protection (compared to 6 months).

**Conclusion:**

When in a pandemic, and while seeking to achieve close to 100% vaccination, understanding these issues pertaining to vaccine acceptability such as effectiveness or duration of protection becomes imperative.

**Supplementary Information:**

The online version contains supplementary material available at 10.1186/s12889-025-25658-w.

## Introduction

The World Health Organization declared the COVID-19 disease as a pandemic on 11 March 2020 [1]. The pharmaceutical industry strived hard to identify agents [drugs, biologics and vaccines] for the treatment and prevention of COVID-19 to mitigate the disease burden [2]. There was a huge surge in vaccine research and development, and the governments across many countries fast-tracked vaccine development and regulatory approval [3]. Despite the introduction of several vaccines subsequently in a very short time span, many of which were made freely available, vaccine uptake was not as brisk as anticipated due to the challenge of vaccine hesitancy [4]. Even prior to COVID-19, India had experienced pockets of vaccine hesitancy. For example, a study in urban slums of Siliguri reported that 83% of families expressed hesitancy toward routine childhood vaccinations, largely due to lack of reliable information and distrust in health services [5]. Similarly, a community-based study in Odisha found around 9% of caregivers of under-five children were hesitant, citing safety concerns and doubts about the necessity of certain vaccines [6]. The government of India had provided all doses of vaccine free of cost based on the government issued identification cards with each primary series dose at least 4 weeks apart starting with the health care workers, frontline workers, elderly and vulnerable, in that order, before providing for the general adult population. Subsequently vaccines were also made available at a subsidized retail price through certain designated private hospitals and medical centres.

Several reasons could explain vaccine hesitancy including concerns regarding the various attributes of the COVID-19 vaccines which include efficacy, side effects and their duration, cost, and country of origin among others [7]. One method which could help governments and policy makers to understand and address vaccine hesitancy is a Discrete Choice Experiment (DCE). A DCE is a quantitative statistical technique that can elicit individual preferences in a large population regarding any goods or services, in this case, a COVID-19 vaccine, wherein the idea of preferences can be broken down into separate characteristics – called “attributes” (as listed earlier)[8, 9].. DCEs evaluating public preferences on COVID-19 vaccines have been reported from China[7, 10–12], USA[10, 13, 14], UK[15], and France [16] but remain limited in Low- and Middle- income countries (LMICs). In India, with its wide cultural and socio-economic diversity, DCEs are particularly useful to understand how individuals trade off vaccine attributes when making decisions. At the time this study was designed and conducted, no DCEs on COVID-19 vaccines had been reported from India. Subsequently, one such study was published[17], but ours is the first large-scale, nationwide DCE, providing comprehensive insights from the general population. The present study was thus envisaged with the primary objective to identify the key attributes of COVID-19 vaccine for acceptability and the participants’ willingness to pay (WTP) for it in the face of an ongoing pandemic by the general population of India. The secondary objectives were to estimate the extent of and predictors of vaccine hesitancy among the Indian public.

## Methods

### Study design and setting

This was a cross-sectional, pan-India survey conducted using the Survey Monkey ™ digital platform after obtaining approval from the Institutional Ethics Committee (EC/OA-135/2021). The survey link was live from 02 October 2021 to 07 December 2021 (a little more than two months) by which time the target sample size was reached. This period corresponded to the time interval between the second wave [Delta] and third wave [Omicron] of the COVID-19 pandemic in India. Per the government of India guidelines at this time point, most health care workers, frontline workers, elderly, and those aged 45 years and above with comorbidities had received the primary series of COVID-19 vaccine. Booster dose of the vaccine was not yet approved.

### Eligibility criteria

All consenting adults of any gender, age 18 years and above who were Indian citizens, and residing in India for a minimum of six months prior to the study initiation were eligible to participate. Non-resident Indians (NRIs), Oversees Citizens of India (OCIs), and those who failed to complete the entire form or failed to answer the trap question correctly (see below for details on trap question) were excluded.

### Study Procedure

*A) Rolling out the survey*: The survey was conducted in two formats: self-administered by participants or assisted by a researcher in a kiosk mode for those who had challenges using an electronic device or were illiterate/semi-literate. Importantly, the survey instrument was exactly the same in both formats, administered through the same electronic platform with no variation in wording, content, or order. In kiosk mode, trained staff provided only technical assistance (such as logging in or navigation), and responses were entered privately by participants. Uniform quality checks, including the trap question, were applied across both modes.

The survey link was disseminated widely via digital platforms (WhatsApp, LinkedIn, Twitter, and email to various societies, groups, organizations, and corporates). In addition, the study team and the Contract Research Organization that assisted with survey development visited public places across India to counsel people and share the link with those willing to participate. The survey was made available in English and five other commonly spoken Indian languages namely Hindi, Marathi, Tamil, Telugu, Kannada and Malayalam. Thus, a non-probability convenient sampling approach was adopted, ensuring inclusion of respondents from all 28 states and 8 union territories of India.

*B) About the link and using it*: On clicking the link, potential participants had access to the participant information sheet and the informed consent document in the language of their choice. Those providing a written informed consent gained access to the survey eligibility form where those who did not fulfil the eligibility criteria were not permitted to respond to the main survey.

The main survey was divided into three sections namely (a) Socio-demographic characteristics including the BG Prasad scale for ascertaining the socio-economic status (SES) class for India modified for 2021 [18] (b) past medical history and COVID- 19 disease and vaccine related history (c) DCE with five pairs of hypothetical vaccines and a sixth trap question (see explanation later). Before the survey went live, it was pilot tested. Data collected during the pilot test was not included in the final analysis.

### The design of the DCE

The DCE in this study was created so as to have five hypothetical vaccine pairs (Vaccine A and Vaccine B) per participant. Each of these pairs were represented by certain pre-decided attributes which were chosen from similar studies found in literature[7, 15, 19]. The attributes in our study were effectiveness, duration of protection, number of injections, common side effects anticipated, risk of severe/serious side effects, place of origin of the vaccine and the cost per injection. Each of these attributes had multiple levels within them. The details of the attributes and their levels used are given in Table 1. Hypothetical vaccine pairs were used instead of already marketed vaccines so that the participants could not be influenced by other factors beyond the attributes listed in this study while they exercised their choice of selecting their most preferred vaccine.

**Table 1:** Attributes and levels

Effectiveness	50% protection 70% protection 90% protection
Duration of protection	6 months 1 year 2 years 5 years
Number of Injections	One Two Three
Common Side effects anticipated	No common side effects. Injection site pain redness and swelling for 1–2 days Fever, body pain for 1–2 days
Risk of severe/serious side effects	1 in one lakh (1 in 1,00,000) 1 in one crore (1 in 1,00,00,000)
Vaccine origin	Indian product Imported product
Cost per injection	Free Rs. 250 Rs. 500 Rs. 1000 Rs. 1500 Rs. 2000

Each of the vaccines (Vaccine A or Vaccine B) had all the attributes mentioned but had just one of the many levels chosen from Table 1 for each of the listed attributes. An example of one such hypothetical vaccine pair is given in Table 2 for ease of understanding.From the many such pairs available, each participant was presented a random set of five vaccine pairs to make an informed choice. (See statistical analysis plan section for more details on how vaccine pairs were created and chosen)

**Table 2:** An example of one hypothetical vaccine pair

Attributes	Vaccine A	Vaccine B
Effectiveness	90%	50%
Duration of protection	5 years	6 months
Number of Injections	One dose	Three doses
Common Side effects anticipated	No common side effects	Fever and body ache 1–2 days
Risk of severe/serious side effects	One in one crore	One in one lakh
Vaccine origin	India	India
Cost per injection	Free	Rs.2000
Which among the above vaccines would you prefer? ▯ Vaccine A ▯ Vaccine B ▯ Neither vaccine/No vaccine		

### Public involvement in this research

The public helped in the data collection of this study by circulating the survey link among their friends and relatives. They also assisted others in their social circle who had difficulty using the online survey tool either due to their educational status or were not comfortable using such digital technology in responding to a survey.

### Sample size

No formal sample-size calculation was performed. Planning was guided by the Johnson–Orme rule-of-thumb for DCEs: *N* ≥ 500×c/(t×a), where c is the maximum number of levels for any attribute (six for cost), t is the number of choice tasks per respondent (five tasks, trap excluded), and a is the number of alternatives per task (two alternates). This suggests a minimum *N* ≈ 300 for main effects. We therefore targeted ~ 10,000 completes to ensure broad national coverage (all 28 states and 8 union territories), obtain precise WTP estimates, support planned subgroup analyses, and accommodate quality exclusions.

### Statistical Analysis Plan:

1. *Prior to data collection*: The principles of conjoint analysis were utilized for the estimations in this study[21, 22]. It is one of the popular survey-experimental methods that estimate respondents’ preferences given their overall evaluations of alternative profiles that vary across multiple attributes[21]. A fully randomized conjoint is a full factorial design and thus, the number of attributes and levels in the current study design would have yielded 2592 unique profiles (3 × 4 × 3 × 3 × 2 × 2 × 6), and the number of unique pair wise choice sets (vaccine pairs) would have been 33,57,936. A total of 40 random pair wise choice sets from 33,57,936 vaccine pairs were constructed using a D-optimality algorithm with the attribute coefficient set to zero. A D-optimal design helps to minimize the covariance of the parameter estimates in the given model[23]. These 40 choice sets were then randomly assigned to eight blocks, each of which had five choice sets. During survey administration, the platform automatically and completely randomized block allocation such that each participant was randomly assigned to one of the eight experimental blocks, with no stratification by demographic or regional variables. The equality of block-wise response distribution was verified post-hoc using a chi-square goodness-of-fit test, which confirmed uniform allocation across all blocks (*p*=.840) (see Additional file 1, Supplementary Table 1) All the five choice sets and the trap question (sixth choice set) were checked by the study team for plausibility and confirmed that no alteration of the attributes was necessary. A direct test is not available to DCEs for their validity and is generally considered to be valid if the WTP estimates are identical to the true WTPs[24]. However, content and construct validity were done with the help of experts from the fields of biostatistics, clinical pharmacology, internal medicine, community medicine, psychologist, social worker and microbiology.
2. *After data collection*: Descriptive statistics were used such as mean and standard deviation for the continuous variables and frequency and proportions for categorical variables. The conditional logit (clogit) model was applied to identify the key attributes of COVID-19 vaccine for its acceptability[22]. In this model, the estimated coefficients represent the relative strength of preference for each attribute level; exponentiating these coefficients yields odds ratios (ORs), which indicate how much more (or less) likely a vaccine profile with that attribute is to be chosen compared to the reference category. The outcomes were thus expressed as OR with 95% Confidence Intervals (CI). In this model, the dependent variable was the respondent’s choice among the three alternatives presented in each choice set while the independent variables were the vaccine attributes and their respective levels. The attribute of ‘cost per injection’ was considered as a continuous variable to facilitate the estimation of willingness to pay (WTP) which was calculated based on the cost attribute in DCE, which provides a monetary value that people place on different attributes of the vaccination program. For example, how much cost that participants are willing to pay to receive a vaccine with 90% protection compared to vaccine with 50% protection was estimated using equation:

$$\begin{aligned}WTP\;(90\%Protection)&=-\frac{\partial\:U/\partial\:\left(90\%Protection\right)}{\partial\:U/\partial\:\left(cost\right)}\\&=\:\:-\frac{\beta\:\left(90\%Protection\right)}{\beta\:\left(cost\right)}\end{aligned}$$

This formula was similarly used to estimate WTP for all other attributes along with 95% confidence intervals (CI). All costs were expressed in Indian Rupees (INR) and United States Dollars (USD) at a conversion rate of one USD = 77 INR (Nov 2021) based on the prevailing exchange rates at the time of study conduct.

To quantify the relative contribution of each vaccine attribute to overall choice, a weighted importance (Relative Attribute Importance, RAI) analysis was conducted post-estimation. For each attribute, the difference between the highest and lowest part-worth utility (coefficient) values across its levels was calculated to obtain the utility range. The RAI for each attribute was then computed as the proportion of its utility range relative to the sum of utility ranges across all attributes, expressed as a percentage. This provides a measure of each attribute’s relative influence on vaccine uptake decisions, with higher RAI values indicating greater importance in respondents’ choice behaviour.

Subgroup analysis was also done to evaluate the association of the public’s preference regarding COVID-19 vaccine attributes for study specific variables such as COVID-19 infection (Present/absent), severity of previous COVID-19 infection (symptomatic/asymptomatic), family history of COVID − 19 (Present/absent), vaccination status (taken two doses – primary series/taken one dose/not taken any dose), Socio-economic status (Upper/middle/lower class), and occupation (healthcare/non-healthcare worker). In response to peer-review feedback, three post-hoc sensitivity analyses were conducted to further examine the robustness of the DCE findings. The first analysis used a subgroup defined as ‘educated guesses’—comprising healthcare workers directly involved in patient interaction and participants with a personal or family history of symptomatic COVID-19. The second analysis used the full dataset including all respondents (*N* = 10,000), while the third was restricted to those who failed the trap question (*N* = 1,241). These additional models were designed to evaluate whether exclusion of trap-failed responses or participant background characteristics materially influenced the preference estimates.

Burden of vaccine hesitancy was expressed as the proportion of respondents choosing the opt-out option of neither vaccine in the choice-sets/vaccine pairs presented to them. The predictors of vaccine hesitancy were identified using the logistic regression (LR) model. Vaccine hesitancy (Dependent Variable) for the purpose of LR analysis was defined as those respondents who responded by choosing the option “neither vaccine” to at least three out of the six vaccine pairs. The independent variables entered into the multivariate LR model included age, sex, marital status, locality (city/town/village), occupation as a healthcare worker, socio-economic status, previous history of COVID-19 infection, presence of commorbidities such as diabetes, hypertension, heart disease, or asthma, and family history of COVID-19 infection. All tests of significance were two sided with an alpha level of 0·05. All the analysis were conducted using STATA software version 16·0 (StataCorp LLC, USA, 2019).

## Results

### Socio-demographic characteristics

A total of *n* = 11,107 participants had consented to participate in the survey of which *n* = 1,107 were excluded (Reasons: *n* = 171 not residing in India in the last 6 months, *n* = 110 were of age less than 18 years and *n* = 826 had submitted incomplete forms). Of the remaining *n* = 10,000 completed forms, *n* = 1241/10,000 (12.41%) failed the trap question and hence were excluded. Thus, the final number analysed was *N* = 8759.

The mean (SD) age was 36·32 (12·61) years, and a greater proportion of the respondents were male (61·03%). The socio-demographic characteristics of the study participants are summarised in Table 3. There were respondents from all the 28 states and the eight union territories of India (see Additional file 1, Supplementary Table 2). A comparison of sociodemographic and health-related characteristics between participants who passed and failed the trap question is shown in Additional file 1, Supplementary Table 3. Respondents who failed the trap question tended to be male, from lower socioeconomic strata, with relatively lower educational attainment, and less likely to have completed COVID-19 vaccination. The distributions of other variables such as marital status, healthcare-worker status, and most comorbidities were broadly similar between the two groups. Detailed history regarding COVID-19 disease, vaccination, personal, and family history is summarized in Additional file 1, Supplementary Table 4.

**Table 3:** Socio-demographic characteristics of the study participants

Variables	Number (*N* = 8759)	Percentage (%)
Gender		
Male	5346	61·03
Female	3411	38·94
Others	2	0·02
Marital status		
Unmarried	2951	33·69
Separated	76	0·87
Widow/Widower	153	1·75
Married and living with spouse	5579	63·69
Locality of living		
City	5100	58·23
Town	2441	27·87
Village or Hamlet	1218	13·91
Education		
Illiterate	142	1·62
Primary School (Up to class 5th Pass)	169	1·93
Middle School (Class 6th, 7th & 8th Pass)	266	3·04
High School (Class 9th & 10th Pass)	498	5·69
Higher Secondary (PUC or Class11th & 12th Pass)	1140	13·02
Diploma/Certificate course	1155	13·19
Degree (UG/PG)	3460	39·50
Professional Degree, Lawyer, Chartered accountant, Engineer, PhD degree holder)	1929	22·02
Health care worker		
Yes	1296	14·80
No	7463	85·20
If health care worker, role (*n* = 1296)		
Doctor	334	25·77
Nurse	98	7·56
Ward boy/sanitary worker/Ward Clerk	43	3·32
Pharmacist	310	23·92
Lab technician	73	5·63
Other role involving patient interaction/patient samples etc.	164	12·65
Office/any other role that does NOT involve interaction with patient/patient samples etc.	274	21·14
Socio Economic Status class		
Upper Class	312	3·56
Upper Middle Class	447	5·10
Middle Class	1496	17·08
Lower Middle Class	2760	31·51
Lower Class	3744	42·74
COVID-19 history		
Personal history positive	1241	14·2
Family history positive	1259	14·4
Status of COVID-19 Vaccination		
Taken both doses	6770	77·3
Taken 1 st dose	1517	17·3
Not taken any dose	472	5·4
Presence of Comorbidities		
Diabetes Mellitus	477	5·4
Hypertension	432	4·9
Heart Problems	190	2·2
Asthma	283	3·2

*SES* Socioeconomic status, *PUC *Pre-University Course, *UG *Undergraduate, *PG *Postgraduate, *PhD *Doctor of Philosophy, *COVID-19 *Coronavirus Disease 2019, *DCE *Discrete Choice Experiment

### Results of the main effects model

The most important attribute based on the main effects model was found to be effectiveness. for A vaccine that gave ‘90% protection’ and ‘70% protection’ (as against 50% protection) had approximately three times (*P* <.001) and two times (*P* <.001) the odds respectively, of being chosen. Duration of protection was the next most important attribute where longer protected duration was more preferred when compared to 6 months and greater preference for 5 years protection (OR = 1.46; *P* <.001) followed by one year protection (OR = 1.40; *P* <.001). In addition, we found that an Indian origin vaccine was preferred over an imported vaccine. (OR = 1.26; *P <* ·001).

As regards safety, there were two attributes: common side effects anticipated and risk of serious/severe side effects. Among these, the preference was least to the vaccines that are likely to cause common local side effects (OR = 0.88, *P <* ·001) when compared to those that cause common systemic side effects (OR = 0.93, *P <* ·001) or severe/serious side effects (OR = 0.94, *P <* ·001). Similarly, the price of the COVID-19 vaccine had a negative and significant effect on the participants, but its coefficient was very small (b = − 0·0006, OR = 0.999, *P <* ·001) suggesting that it was not considered as important as the other attributes.

As regards WTP, participants were willing to pay an additional INR 1549 (20·12 USD) for a vaccine offering 90% protection compared to one offering 50% protection, and an additional INR 587 (7·62 USD) for a vaccine offering 5 years of protection compared to one lasting 6 months. For vaccines with adverse effects, the participants were willing to pay lesser than those which did not have the common adverse effects or have the least likelihood of having the severe/serious adverse effects. These results are summarised in Table 4. In the RAI analysis, vaccine effectiveness had the highest RAI (54.2%), followed by duration of protection (20.6%). Other attributes such as vaccine origin (12.6%), common side effects (6.6%), risk of severe or serious side effects (3.4%), and number of injections (2.6%) contributed comparatively less to respondents’ vaccine choice decisions.

**Table 4:** Main effects model and willingness to pay

Attributes (*N* = 8759)		Main Effects Model				Willingness to Pay (INR)	
		Coefficient (SE)	Odds Ratio	95% CI	*P* value	Coefficient (SE)	95% CI
Effectiveness	50% protection	-	-	-	-	-	-
	70% protection	0·658 (0·017)	1.93	1.87–2.00	< 0·001	1013·065 (29·53)	955·186–1070·943
	90% protection	1·005 (0·018)	2.73	2.64–2.83	< 0·001	1548·661 (35·964)	1478·173–1619·149
Duration of protection	6 months	-	-	-	-	-	-
	1 year	0·335 (0·022)	1.40	1.34–1.46	< 0·001	515·634 (34·224)	448·557–582·712
	2 years	0·225 (0·02)	1.25	1.20–1.30	< 0·001	346·720 (31·833)	284·329–409·112
	5 years	0·381 (0·02)	1.46	1.41–1.52	< 0·001	586·542 (31·558)	524·69–648·394
Number of Injections	Three times	-	-	-	-	-	-
	One time	0·049 (0·017)	1.05	1.02–1.09	0·004	75·289 (26·436)	23·474–127·103
	Two times	0·045 (0.017)	1.05	1.01–1.08	0·009	69·370 (26·735)	16·971 − 121·769
Vaccine origin	Imported	-	-	-	-	-	-
	Indian	0·233 (0·012)	1.26	1.23–1.29	< 0·001	359·560 (19·303)	321·728 − 397·392
Common side effects anticipated	No side effects	-	-	-	-	-	-
	Injection site pain redness and swelling for 1–2 days	−0·123 (0·017)	0.88	0.86–0.91	< 0·001	−188·970 (25·721)	−239·382 - (−138·558)
	Fever, body pain for 1–2 days	−0·073 (0·017)	0.93	0.90–0.96	< 0·001	−112·751 (26·196)	−164·094 - (−61·407)
Risk of severe/serious side effect	One in one crore (10 million)	-	-	-	-	-	-
	One in one lakh (100 thousand)	−0·063 (0·012)	0.94	0.92–0.96	< 0·001	−96·488 (18·878)	−133·487 - (−59·488)
Cost per injection		−0·0006 (0·00001)	0.999	0.999–0.999	< 0·001	-	-

### Results of the subgroup analysis

A COVID-19 vaccine with a higher effectiveness was more likely to yield a higher coefficient denoting higher WTP and greater importance given by those with previous personal/family history of COVID-19, those with symptomatic disease, those who had taken two doses of vaccine (> taken one dose > no doses), lower SES (> middle SES > upper SES) and by healthcare workers. Healthcare workers were willing to spend an additional INR 3736 (48.52 USD) when compared to non-healthcare workers who were willing to spend only an additional INR 1357 (17.62 USD) for a 90% effective vaccine. A similar trend was seen for the duration of protection being longer i.e., 5 years *(versus* 6 months). Detailed results of the sub-group analyses are given in Additional File 1, Supplementary Tables 5–10.

### Results of the sensitivity analysis

The ‘educated-guesses’ subgroup produced results closely aligned with the main analysis (see Additional file 1, Supplementary Table 11). Similarly, the full-dataset model yielded estimates that were almost identical in direction and magnitude to those from the trap-passed analysis, indicating that the inclusion of failed-trap responses did not meaningfully alter findings. In contrast, the model limited to the trap-failed group produced small, statistically non-significant, and directionally inconsistent coefficients (see Additional file 1, Supplementary Tables 12 and 13), consistent with inattentive or random responding.

### Vaccine hesitancy

**Fig. 1:**
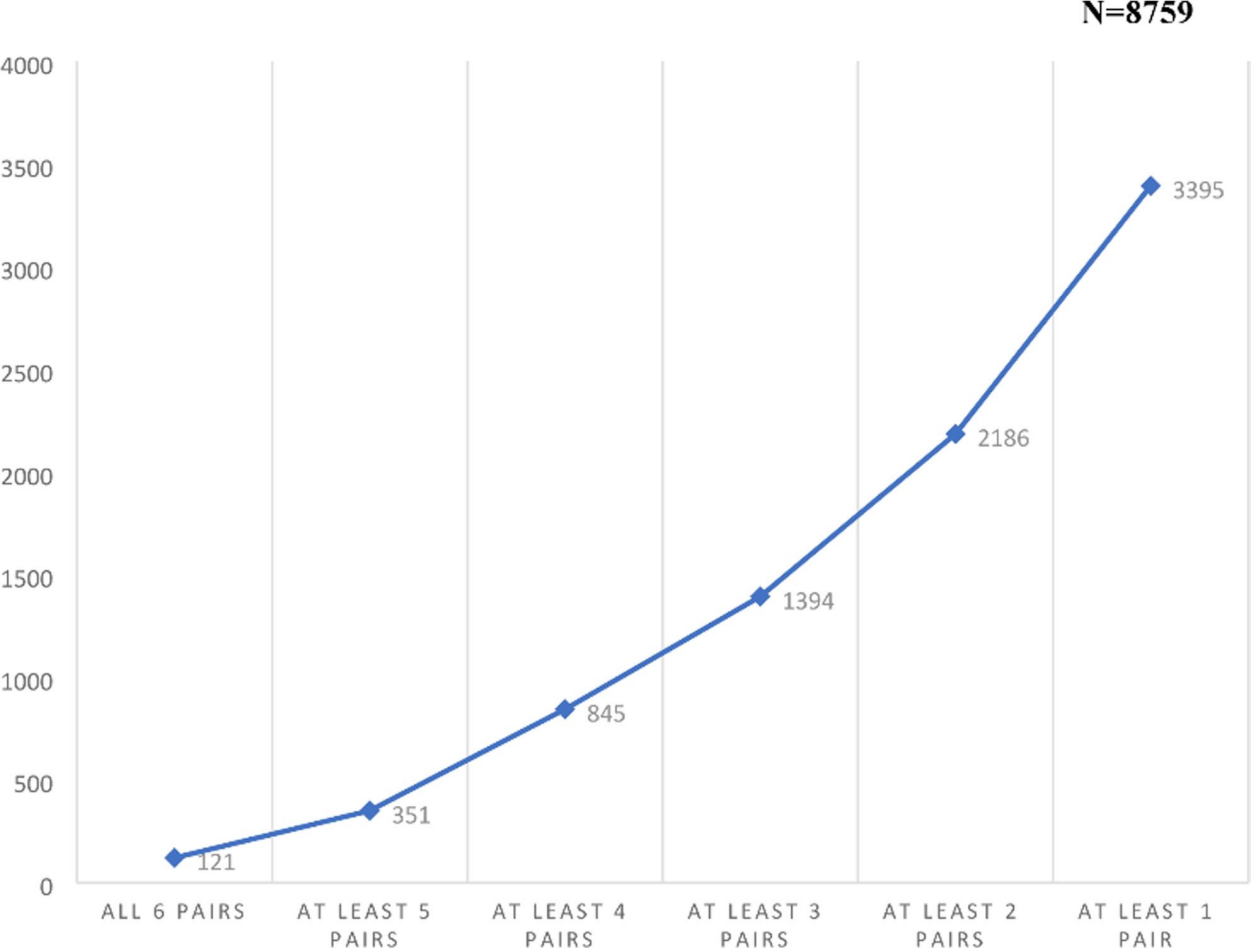
Cumulative proportion of respondents choosing opt-out option denoting vaccine hesitancy

Among a total of n=8759 respondents, n=121 (1.4%) chose the opt-out option for all the six vaccine pairs and n = 1394 (15.92%) chose for at least 3/6 vaccine pairs. A graph showing cumulative proportion of participants choosing the opt-out option is given in Figure 1. The choice of the total number of vaccine pairs attempted by the participants were n = 52554 (8759 participants * six vaccine pairs), of which n = 8292 vaccines pairs (15.78%) were responded as neither vaccine preferred within the given pair. The results of the univariate and multivariate analyses identifying the predictors of vaccine hesitancy are given in Table 5. The significant predictors [adjusted Odds Ratio (95% Confidence Interval lower limit, upper limit); *P* value] were male sex (men relative to women) [1·17 (1·04, 1·33); *P = *·011], upper [1·89 (1·37, 2·60); *P <* ·001], and middle socio-economic class [1·91 (1·68, 2·17); *P <*·001] (relative to lower socio-economic class), and presence of comorbidities such as diabetes [1·34 (1·06, 1·69); *P*= ·014,] cardiac illnesses [1·44 (1·02, 2·02); *P = *·038], or asthma [2·34 (1·80, 3·05); *P <* ·001]. City dwellers [0·64 (0·57, 0·72); *P <* ·001], and those with previous medical history [0·82 (0·68, 1·00); *P =* ·048], or family history of COVID-19 infection [0·70 (0·57, 0·86); *P =* ·001] were least likely to be hesitant. Marital status, hypertension, and being a healthcare worker, did not predict vaccine hesitancy.

**Table 5:** Univariate and multivariate logistic regression analysis for vaccine hesitancy

Variables (*N* = 8759)		Vaccine Hesitancy - At least three pair		Univariate Analysis		Multivariate Analysis	
		Yes (*n* = 1394)	No (*n* = 7365)	OR (95% CI)	*P* value	aOR (95% CI)	*P* value
		*n* (%)	*n* (%)				
Age *(Mean ± SD)*		39·14 ± 12·7	35·79 ± 12·53	1·02 (1·02, 1·02)	**< 0·001**	1·02 (1·01, 1·02)	**< 0·001**
Sex	Male	924 (66·4)	4422 (60)	1·31 (1·16, 1·48)	**< 0·001**	1·17 (1·04, 1·33)	**0·011**
	Female	468 (33·6)	2943 (40)	1·00		1·00	
Marital status	Married and living with spouse	979 (70·2)	4600 (62·5)	1·42 (1·25, 1·61)	**< 0·001**	1·11 (0·96, 1·29)	0·169
	Unmarried/Separated/Widow	415 (29·8)	2765 (37·5)	1·00		1·00	
Locality	City	654 (46·9)	4446 (60·4)	0.58 (0·52, 0·65)	**< 0·001**	0·64 (0·57, 0·72)	**< 0·001**
	Town/Village	740 (53.1)	2919 (39.6)	1.00		1.00	
Health care worker	Yes	140 (10)	1156 (15·7)	0·60 (0·50, 0·72)	**< 0·001**	0·85 (0·70, 1·04)	0·113
	No	1254 (90)	6209 (84·3)	1·00		1.00	
Socio-economic status	Upper class	53 (3·8)	259 (3·5)	1·64 (1·20, 2·24)	**0·002**	1.89 (1·37, 2·60)	**< 0·001**
	Middle class	926 (66·4)	3777 (51·3)	1·97 (1·74, 2·23)	**< 0·001**	1·91 (1·68, 2·17)	**< 0·001**
	Lower class	415 (29·8)	3329 (45·2)	1·00		1·00	
Previous COVID-19 infection	Yes	152 (10·9)	1089 (14·8)	0·71 (0·59, 0·84)	**< 0·001**	0·82 (0·68, 1·00)	**0·048**
	No	1242 (89·1)	6276 (85·2)	1·00		1·00	
Diabetes	Yes	112 (8)	365 (5)	1·68 (1·34, 2·09)	**< 0·001**	1·34 (1·06, 1·69)	**0·014**
	No	1282 (92)	7000 (95)	1·00		1·00	
Hypertension	Yes	61 (4·4)	371 (5)	0·86 (0·65, 1·14)	0·296	-	-
	No	1333 (95·6)	6994 (95)	1·00			
Heart Problem	Yes	53 (3·8)	137 (1·9)	2·09 (1·51, 2·88)	**< 0·001**	1·44 (1·02, 2·02)	**0·038**
	No	1341 (96·2)	7228 (98·1)	1·00		1·00	
Asthma	Yes	95 (6·8)	188 (2·6)	2·79 (2·17, 3·60)	**< 0·001**	2·34 (1·80, 3·05)	**< 0·001**
	No	1299 (93·2)	7177 (97·4)	1·00		1·00	
Family History of COVID-19	Yes	137 (9·8)	1122 (15·2)	0·61 (0·50, 0·73)	**< 0·001**	0·70 (0·57, 0·86)	**0·001**
	No	1257 (90·2)	6243 (84·8)	1·00		1·00	

With regards to the perceived difficulty level of the questionnaire, only *n* = 311/8759 (3·6%) expressed that they found it ‘very hard’ to respond to the questionnaire. Of the remaining, *n* = 2487/8759 (28·4%) perceived it to be very easy, *n* = 2981 (34·0) as easy, *n* = 1971/8759 as neutral, and *n* = 1007/8759 (11·5%) as hard.

## Discussion

We conducted a nation-wide DCE (online and offline mode) to identify the most preferred COVID-19 vaccine attributes/characteristics, their WTP and their trade-offs. We found that, in India, the most important attribute was better effectiveness (90%), followed by longer duration of protection (5 years) and Indian origin of the vaccine. A more expensive vaccine was less preferred (relative to the less expensive ones) although, cost per se while choosing a vaccine was a least preferred attribute. With regards to WTP, the respondents were willing to incur an additional expenditure of INR 1549 (20 USD) to obtain a 90% effective vaccine (relative to one that was 50% effective).

A vaccine that was highly effective was the single most important, preferred attribute followed by longer duration of protection and lesser side effects indicating that in a pandemic, effectiveness, and duration of protection takes priority over all other attributes as it enables a faster return to one’s routine and possibly protects the family as well. These findings are in line with findings from other similar studies from other countries such as the UK[15], USA[13, 19], and China [10] indicating that in a pandemic with great human suffering the thinking does not differ between an HMIC and an LMIC. Further, Indians preferred an India-made vaccine. This is similar to that reported by Kreps et al. from the USA, where people preferred a vaccine manufactured in their own country. [19] The results of this current study are also in line with another study with a smaller size (*N*= 1371, five Indian states only) who have reported that a highly effective, domestically developed vaccine with lesser side effects is likely to improve vaccine uptake[17]. This shows that Indians have significant confidence in the vaccines manufactured within the country and this confidence is likely to have been boosted by the frequent media reports of India being a major vaccine manufacturer and exporter and Indian vaccines (both COVID and non-COVID) have been exported to many other nations for several years [25]. 

Our findings carry important implications for public health and vaccine policy in India. Communication strategies should prioritise vaccine effectiveness and durability of protection, as these were the strongest drivers of acceptance. Emphasising vaccines manufactured in India may be leveraged to build public trust, in line with evidence from other countries showing higher acceptance of domestically produced vaccines[7, 19]. The willingness-to-pay estimates provide insights for policy on pricing and subsidies, particularly in private sector distribution where vaccines are offered at a cost. Moreover, the differential preferences observed across socio-economic strata underscore the need for tailored messaging and targeted outreach to ensure equitable vaccine uptake. Together, these implications can inform both routine immunisation strategies and preparedness for future pandemics.

Vaccine hesitancy was seen in approximately one-quarter of the participants indicating that this is an important issue for policy makers to address. In our sample, hesitancy was more common among men, those from middle and upper socio-economic classes, and individuals with comorbidities such as diabetes, cardiac illness, or asthma. The higher hesitancy among men may reflect gender-based differences in risk perception, while the association with middle and upper socio-economic classes could relate to greater access to conflicting information or stronger personal preferences regarding vaccination. Individuals with comorbidities may be more concerned about adverse effects, which could explain their increased hesitancy. Conversely, urban residents and participants with a personal or family history of COVID-19 infection were less likely to be hesitant, likely reflecting greater awareness of disease risk and higher exposure to credible health information. These findings highlight how both socio-demographic and health-related factors shape hesitancy. Addressing such hesitancy requires multipronged strategies: targeted risk communication addressing safety concerns among people with comorbidities, tailored outreach for men and higher socio-economic groups, and leveraging the positive influence of lived experience with COVID-19 through survivor advocacy and community engagement.

Vaccine hesitancy is a spectrum where reasons include but are not limited to, lack of confidence about the vaccine candidates and the public system, misinformation, distrust, inappropriate risk perception about COVID-19 disease, and more importantly concerns regarding the various vaccine attributes such as its effectiveness and risk of adverse effects[4]. Our DCE assessed preferences based on clearly defined vaccine attributes, but in practice hesitancy is also strongly shaped by misinformation and distrust. Thus, while stated preferences may not fully capture behaviours influenced by rumour, our findings complement existing research by quantifying the relative weight of objective vaccine features. These observations align with prior experiences, such as the 2009 H1N1 influenza pandemic, where similar reasons were cited for vaccine refusal[28]. This suggests that more than a decade after the last pandemic and despite a strikingly high mortality rate in comparison to the last pandemic, nations of the world have still not taken adequate efforts to address the issue of vaccine hesitancy among general public especially in a pandemic setting[29].

The main strength of our study lies in the fact that our sample size was large, and it was able to capture the perceptions of almost the entire country with a good representation of different subgroups of all the demographic characteristics. India is the seventh largest country in the world by geographical area and the second most populous with diverse cultural, religious and traditional backgrounds and a nation-wide survey is likely to be both representative and generalizable to populations living in other LMICs.

Our study is however limited by non-probability sampling and use of the hybrid mode, thereby making it difficult to estimate the response rate and fully mitigate potential selection bias. Dissemination through digital platforms and organisational mailing lists may have biased recruitment towards urban and digitally literate groups, although kiosk administration was included to improve inclusivity. Additionally, comparison between those who passed and failed the trap question revealed certain demographic differences. These differences, which align with trends reported in other online DCEs, suggest that exclusion of trap-fail respondents may have modestly improved data quality by retaining participants with greater engagement and comprehension. However, this exclusion could have slightly reduced the representation of lower-socioeconomic or lower-education groups, which should be considered when interpreting the generalizability of the findings. A key strength of this study is the inclusion of multiple post-hoc sensitivity analyses performed in response to peer-review feedback. The consistent results across the full dataset, the ‘educated-guesses’ subgroup, and the trap-failed subset confirm the robustness and internal validity of the DCE and indicate that exclusion of inattentive responses did not bias the study’s conclusions. Also, although the vaccine pairs in the DCE were stated as being hypothetical, COVID-19 vaccines were constantly discussed in the media and therefore there exists a small possibility that the respondents linked one or more of the given attributes to the existing commercially available COVID-19 vaccines and chose options considering other attributes as well that were not a part of this study. Finally, there is a likelihood that the vaccine hesitancy burden may not be accurate as compared to the traditional methods of assessing the burden of vaccine hesitancy as they could have been influenced by the hypothetical attributes and levels listed. Also, we had approximately 8% non-responders and this could have been related to the proposed outcomes as well. However, we believe that the large sample size would have mitigated these biases as well.

## Supplementary Information

Additional file 1: Supplementary tables for the COVID-19 Vaccine Discrete Choice Experiment (DCE) study.

## Data Availability

The dataset analysed during the current study is available in the Harvard Dataverse repository, at URL: https:/doi.org/10.7910/DVN/ZYVSIA.
