# Supplementary material for: Prediction of factors influencing adults‘ likelihood of accepting any COVID-19 vaccination and their willingness to pay – a nation-wide discrete choice experiment among the general public from India
Source: BMC Public Health. 2025 Dec 30;25:4369. doi: 10.1186/s12889-025-25658-w (PMC12754888; doi:10.1186/s12889-025-25658-w)
Supplement: Supplementary file 1 — Additional file 1: Supplementary tables for the COVID-19 Vaccine Discrete Choice Experiment (DCE) study. [file 12889_2025_25658_MOESM1_ESM.docx]

*Supplementary material*

Prediction of factors influencing adults‘ likelihood of accepting any COVID-19 vaccination and their willingness to pay – a nation-wide discrete choice experiment from India.

*Supplementary Table 1:Blockwise distribution of responses*

| **Blocks** | **Overall (N = 10000) (P-Value = 0.840)** | | **Trap passed (N = 8759) (P-Value = 0.886)** | | **Trap failed (N = 1241) (P-Value = 0.851)** | |
| --- | --- | --- | --- | --- | --- | --- |
|  | **Freq** | **%** | **Freq** | **%** | **Freq** | **%** |
| 1 | 1259 | 12.59 | 1119 | 12.78 | 140 | 11.28 |
| 2 | 1242 | 12.42 | 1085 | 12.39 | 157 | 12.65 |
| 3 | 1292 | 12.92 | 1129 | 12.89 | 163 | 13.13 |
| 4 | 1247 | 12.47 | 1092 | 12.47 | 155 | 12.49 |
| 5 | 1248 | 12.48 | 1089 | 12.43 | 159 | 12.81 |
| 6 | 1263 | 12.63 | 1100 | 12.56 | 163 | 13.13 |
| 7 | 1246 | 12.46 | 1086 | 12.40 | 160 | 12.89 |
| 8 | 1203 | 12.03 | 1059 | 12.09 | 144 | 11.60 |

*Supplementary Table 2: State-wise distribution of respondents (N=8759)*

| *Sl No* | *State/ Union territories* | *Number* | *Percentage* |
| --- | --- | --- | --- |
| 1 | Andaman and Nicobar Islands (UT) | 2 | 0·02 |
| 2 | Andhra Pradesh | 380 | 4·34 |
| 3 | Arunachal Pradesh | 31 | 0·35 |
| 4 | Assam | 773 | 8·83 |
| 5 | Bihar | 68 | 0·78 |
| 6 | Chandigarh (UT) | 22 | 0·25 |
| 7 | Chhattisgarh | 4 | 0·05 |
| 8 | Dadra and Nagar Haveli and Daman and Diu (UT) | 1 | 0·01 |
| 9 | Delhi (UT) | 387 | 4·42 |
| 10 | Goa | 2 | 0·02 |
| 11 | Gujarat | 1588 | 18·13 |
| 12 | Haryana | 72 | 0·82 |
| 13 | Himachal Pradesh | 45 | 0·51 |
| 14 | Jammu and Kashmir (UT) | 18 | 0·21 |
| 15 | Jharkhand | 14 | 0·16 |
| 16 | Karnataka | 266 | 3·04 |
| 17 | Kerala | 464 | 5·30 |
| 18 | Ladakh (UT) | 3 | 0·03 |
| 19 | Lakshadweep (UT) | 1 | 0·01 |
| 20 | Madhya Pradesh | 387 | 4·42 |
| 21 | Maharashtra | 1399 | 15·97 |
| 22 | Manipur | 23 | 0·26 |
| 23 | Meghalaya | 16 | 0·18 |
| 24 | Mizoram | 22 | 0·25 |
| 25 | Nagaland | 18 | 0·21 |
| 26 | Odisha | 28 | 0·32 |
| 27 | Pondicherry (UT) | 9 | 0·10 |
| 28 | Punjab | 21 | 0·24 |
| 29 | Rajasthan | 565 | 6·45 |
| 30 | Sikkim | 5 | 0·06 |
| 31 | Tamil Nadu | 258 | 2·95 |
| 32 | Telangana | 212 | 2·42 |
| 33 | Tripura | 2 | 0·02 |
| 34 | Uttar Pradesh | 151 | 1·72 |
| 35 | Uttarakhand | 30 | 0·34 |
| 36 | West Bengal | 1472 | 16·81 |

* UT – Union Territory

*Supplementary Table 3: Comparison of sociodemographic and health-related characteristics between participants who passed and failed the trap question*

| **Variables** | **Overall (N = 10000)** | | **Trap Passed (N = 8759)** | | **Trap Failed (N = 1241)** | | **P-value** |
| --- | --- | --- | --- | --- | --- | --- | --- |
|  | **Freq** | **%** | **Freq** | **%** | **Freq** | **%** |  |
| **Gender** |  |  |  |  |  |  |  |
| Male | 6158 | 61.58 | 5346 | 61·03 | 812 | 65.43 | 0.010 |
| Female | 3840 | 38.40 | 3411 | 38·94 | 429 | 34.57 |  |
| Others | 2 | 0.02 | 2 | 0·02 | 0 | 0.00 |  |
| **Marital status** |  |  |  |  |  |  |  |
| Unmarried | 3335 | 33.35 | 2951 | 33·69 | 384 | 30.94 | 0.200 |
| Separated | 84 | 0.84 | 76 | 0·87 | 8 | 0.64 |  |
| Widow/ Widower | 177 | 1.77 | 153 | 1·75 | 24 | 1.93 |  |
| Married and living with spouse | 6404 | 64.04 | 5579 | 63·69 | 825 | 66.48 |  |
| **Locality of living** |  |  |  |  |  |  |  |
| City | 5775 | 57.75 | 5100 | 58·23 | 675 | 54.39 | 0.037 |
| Town | 2817 | 28.17 | 2441 | 27·87 | 376 | 30.30 |  |
| Village or Hamlet | 1408 | 14.08 | 1218 | 13·91 | 190 | 15.31 |  |
| **Education** |  |  |  |  |  |  |  |
| Illiterate | 169 | 1.69 | 142 | 1.62 | 27 | 2.18 | <0.001 |
| Primary School (Up to class 5th Pass) | 183 | 1.83 | 169 | 1.93 | 14 | 1.13 |  |
| Middle School (Class 6th, 7th & 8th Pass) | 305 | 3.05 | 266 | 3.04 | 39 | 3.14 |  |
| High School (Class 9th & 10th Pass) | 561 | 5.61 | 498 | 5.69 | 63 | 5.08 |  |
| Higher Secondary (PUC or Class11th & 12th Pass) | 1394 | 13.94 | 1140 | 13.02 | 254 | 20.47 |  |
| Diploma / Certificate course | 1354 | 13.54 | 1155 | 13.19 | 199 | 16.04 |  |
| Degree (UG / PG) | 3903 | 39.03 | 3460 | 39.5 | 443 | 35.7 |  |
| Professional Degree, Lawyer, Chartered accountant, Engineer, PhD degree holder) | 2131 | 21.31 | 1929 | 22.02 | 202 | 16.28 |  |
| **Health care worker** |  |  |  |  |  |  |  |
| Yes | 1460 | 14.60 | 1296 | 14.80 | 164 | 13.22 | 0.152 |
| No | 8540 | 85.40 | 7463 | 85.20 | 1077 | 86.78 |  |
| **If health care worker, role** |  |  |  |  |  |  |  |
| Doctor | 357 | 24.45 | 334 | 25.77 | 23 | 14.02 | 0.005 |
| Nurse | 116 | 7.95 | 98 | 7.56 | 18 | 10.98 |  |
| Ward boy/ sanitary worker/ Ward Clerk | 52 | 3.56 | 43 | 3.32 | 9 | 5.49 |  |
| Pharmacist | 348 | 23.84 | 310 | 23.92 | 38 | 23.17 |  |
| Lab technician | 80 | 5.48 | 73 | 5.63 | 7 | 4.27 |  |
| Other role involving patient interaction/ patient samples etc | 183 | 12.53 | 164 | 12.65 | 19 | 11.59 |  |
| Office /any other role that does NOT involve interaction with patient/patient samples etc | 324 | 22.19 | 274 | 21.14 | 50 | 30.49 |  |
| **Socio Economic Status class** |  |  |  |  |  |  |  |
| Upper Class | 4148 | 41.48 | 3744 | 42.74 | 404 | 32.55 | <0.001 |
| Upper Middle Class | 3148 | 31.48 | 2760 | 31.51 | 388 | 31.27 |  |
| Middle Class | 1741 | 17.41 | 1496 | 17.08 | 245 | 19.74 |  |
| Lower Middle Class | 567 | 5.67 | 447 | 5.1 | 120 | 9.67 |  |
| Lower Class | 396 | 3.96 | 312 | 3.56 | 84 | 6.77 |  |
| **COVID-19 history** |  |  |  |  |  |  |  |
| Personal history positive | 1391 | 13.91 | 1241 | 14.17 | 150 | 12.09 | 0.052 |
| Family history positive | 1399 | 13.99 | 1259 | 14.37 | 140 | 11.28 | 0.004 |
| **Status of COVID-19 Vaccination** |  |  |  |  |  |  |  |
| Taken both doses | 7614 | 76.14 | 6770 | 77.29 | 844 | 68.01 | <0.001 |
| Taken 1st dose | 1850 | 18.5 | 1517 | 17.32 | 333 | 26.83 |  |
| Not taken any dose | 536 | 5.36 | 472 | 5.39 | 64 | 5.16 |  |
| **Presence of Comorbidities** |  |  |  |  |  |  |  |
| Diabetes Mellitus | 534 | 5.34 | 477 | 5.45 | 57 | 4.59 | 0.237 |
| Hypertension | 467 | 4.67 | 432 | 4.93 | 35 | 2.82 | 0.001 |
| Heart Problems | 206 | 2.06 | 190 | 2.17 | 16 | 1.29 | 0.053 |
| Asthma | 305 | 3.05 | 283 | 3.23 | 22 | 1.77 | 0.007 |

SES – Socioeconomic status; PUC – Pre-University Course; UG – Undergraduate; PG – Postgraduate; PhD – Doctor of Philosophy; COVID-19 – Coronavirus Disease 2019; DCE – Discrete Choice Experiment

*Supplementary Table 4: History regarding COVID-19 disease, vaccination, personal and family history (N = 8759)*

| **Variable Name** | **Category** | **Overall (N = 10000)** | | **Trap passed (N = 8759)** | | **Trap failed (N = 1241)** | | **P-Value** |
| --- | --- | --- | --- | --- | --- | --- | --- | --- |
|  |  | **Freq** | **%** | **Freq** | **%** | **Freq** | **%** |  |
| *Were you ever diagnosed with COVID-19 infection?* | Yes | 1391 | 13.91 | 1241 | 14.17 | 150 | 12.09 | 0.052 |
|  | No | 8609 | 86.09 | 7518 | 85.83 | 1091 | 87.91 |  |
| If you have been diagnosed with COVID-19 infection, how many times were you diagnosed with COVID-19? | 1 time | 1246 | 89.58 | 1116 | 89.93 | 130 | 86.67 | 0.143 |
|  | 2 times | 133 | 9.56 | 116 | 9.35 | 17 | 11.33 |  |
|  | 3 times | 12 | 0.86 | 9 | 0.73 | 3 | 2.00 |  |
|  | 4 or more times | 0 | 0.00 | 0 | 0.00 | 0 | 0.00 |  |
| What was the severity of your COVID-19 infection? [If diagnosed more than once, mark the option that correspond to the worst ever severity | Asymptomatic | 452 | 32.49 | 418 | 33.68 | 34 | 22.67 | 0.020 |
|  | Home isolation with symptoms but no oxygen requirement | 731 | 52.55 | 648 | 52.22 | 83 | 55.33 |  |
|  | Home Isolation with symptoms and required oxygen | 62 | 4.46 | 50 | 4.03 | 12 | 8.00 |  |
|  | Hospitalization without oxygen requirement | 106 | 7.62 | 92 | 7.41 | 14 | 9.33 |  |
|  | Hospitalization in ward with oxygen requirement | 30 | 2.16 | 25 | 2.01 | 5 | 3.33 |  |
|  | Hospitalization in ICU without ventilator | 4 | 0.29 | 3 | 0.24 | 1 | 0.67 |  |
|  | Hospitalization in ICU with ventilator | 6 | 0.43 | 5 | 0.40 | 1 | 0.67 |  |
| Severity of Covid-19 infection (Recoded) | Asymptomatic | 452 | 32.49 | 418 | 33.68 | 34 | 22.67 | 0.009 |
|  | Symptomatic | 939 | 67.51 | 823 | 66.32 | 116 | 77.33 |  |
| *What is the status of your COVID-19 vaccination as of today?* | Taken both doses | 7614 | 76.14 | 6770 | 77.29 | 844 | 68.01 | <0.001 |
|  | Taken 1st dose | 1850 | 18.5 | 1517 | 17.32 | 333 | 26.83 |  |
|  | Not taken any dose | 536 | 5.36 | 472 | 5.39 | 64 | 5.16 |  |
| *Diabetes* | Yes | 534 | 5.34 | 477 | 5.45 | 57 | 4.59 | 0.237 |
|  | No | 9466 | 94.7 | 8282 | 94.6 | 1184 | 95.41 |  |
| *Hypertension* | Yes | 467 | 4.67 | 432 | 4.93 | 35 | 2.82 | 0.001 |
|  | No | 9533 | 95.33 | 8327 | 95.07 | 1206 | 97.18 |  |
| *Heart Problem* | Yes | 206 | 2.06 | 190 | 2.17 | 16 | 1.29 | 0.053 |
|  | No | 9794 | 97.9 | 8569 | 97.8 | 1225 | 98.71 |  |
| *Asthma* | Yes | 305 | 3.05 | 283 | 3.23 | 22 | 1.77 | 0.007 |
|  | No | 9695 | 97 | 8476 | 96.8 | 1219 | 98.23 |  |
| *Did you have anyone else in the family who was diagnosed with COVID-19?* | Yes | 1399 | 14 | 1259 | 14.4 | 140 | 11.28 | 0.004 |
|  | No | 8601 | 86 | 7500 | 85.6 | 1101 | 88.72 |  |
| If yes, no. of relative who were diagnosed with COVID-19 (n=1259) | 1 | 677 | 48.39 | 600 | 47.66 | 77 | 55.00 | 0.283 |
|  | 2 | 424 | 30.31 | 383 | 30.42 | 41 | 29.29 |  |
|  | 3 | 152 | 10.86 | 139 | 11.04 | 13 | 9.29 |  |
|  | 4 | 71 | 5.08 | 68 | 5.40 | 3 | 2.14 |  |
|  | 5 | 35 | 2.50 | 32 | 2.54 | 3 | 2.14 |  |
|  | 6 | 16 | 1.14 | 16 | 1.27 | 0 | 0.00 |  |
|  | 7 | 7 | 0.50 | 7 | 0.56 | 0 | 0.00 |  |
|  | 8 | 3 | 0.21 | 3 | 0.24 | 0 | 0.00 |  |
|  | 9 | 2 | 0.14 | 2 | 0.16 | 0 | 0.00 |  |
|  | 10 or more | 12 | 0.86 | 9 | 0.71 | 3 | 2.14 |  |
| *Was there anyone in your family who died because of COVID-19?* | Yes | 193 | 13.80 | 172 | 13.66 | 21 | 15.00 | 0.759 |
|  | No | 1206 | 86.20 | 1087 | 86.34 | 119 | 85.00 |  |
| If yes, how many of them lost their lives because of COVID-19? | 1 | 157 | 81.35 | 141 | 81.98 | 16 | 76.19 | 0.355 |
|  | 2 | 26 | 13.47 | 23 | 13.37 | 3 | 14.29 |  |
|  | 3 | 6 | 3.11 | 5 | 2.91 | 1 | 4.76 |  |
|  | 4 | 2 | 1.04 | 1 | 0.58 | 1 | 4.76 |  |
|  | 5 | 2 | 1.04 | 2 | 1.16 | 0 | 0.00 |  |
| *Was there anyone in your family who was admitted in ICU for COVID-19 and recovered?* | Yes | 215 | 15.37 | 198 | 15.73 | 17 | 12.14 | 0.321 |
|  | No | 1184 | 84.63 | 1061 | 84.27 | 123 | 87.86 |  |
| If yes, how many of your family members in the household who were admitted in ICU for COVID-19 and recovered? | 1 | 165 | 76.74 | 154 | 77.78 | 11 | 64.71 | 0.552 |
|  | 2 | 41 | 19.07 | 35 | 17.68 | 6 | 35.29 |  |
|  | 3 | 2 | 0.93 | 2 | 1.01 | 0 | 0.00 |  |
|  | 4 | 2 | 0.93 | 2 | 1.01 | 0 | 0.00 |  |
|  | 5 | 2 | 0.93 | 2 | 1.01 | 0 | 0.00 |  |
|  | 6 or more | 3 | 1.40 | 3 | 1.52 | 0 | 0.00 |  |

*Supplementary Table 5A: Presence of personal history of COVID-19 infection (n=1241)*

| *Attributes* | |  | *Main Effects Model* | | | | *Willingness to Pay (INR)* | |
| --- | --- | --- | --- | --- | --- | --- | --- | --- |
|  |  | *Coefficient (SE)* | *Odds Ratio* | *95% CI* | *P value* | *Coefficient (SE)* | | *95% CI* |
| Effectiveness | 50% protection | - | - | - | - | - | | - |
|  | 70% protection | 0.736 (0.044) | 2.09 | 1.91 - 2.28 | *<0.001* | 1476.825 (116.397) | | 1248.692 - 1704.957 |
|  | 90% protection | 1.181 (0.049) | 3.26 | 2.96 - 3.58 | *<0.001* | 2371.392 (159.825) | | 2058.141 - 2684.643 |
| Duration of protection | 6 months | - | - | - | *-* | - | | - |
|  | 1 year | 0.446 (0.057) | 1.56 | 1.40 - 1.75 | *<0.001* | 895.239 (121.019) | | 658.046 - 1132.433 |
|  | 2 years | 0.411 (0.053) | 1.51 | 1.36 - 1.68 | *<0.001* | 825.93 (115.056) | | 600.424 - 1051.436 |
|  | 5 years | 0.494 (0.052) | 1.64 | 1.48 - 1.81 | *<0.001* | 992.552 (114.889) | | 767.373 - 1217.73 |
| Number of Injections | Three times | - | - | - | - | - | | - |
|  | One time | -0.004 (0.044) | 0.996 | 0.91 - 1.09 | 0.920 | -8.953 (89.073) | | -183.532 - 165.626 |
|  | Two times | 0.045 (0.045) | 1.05 | 0.96 - 1.14 | 0.323 | 89.614 (91.013) | | -88.768 - 267.995 |
| Side effects anticipated | No side effects | - | - | - | - | - | | - |
|  | Injection site pain redness and swelling for 1-2 days | -0.094 (0.043) | 0.91 | 0.84 - 0.99 | *0.031* | -187.805 (87.387) | | -359.081 - (-16.529) |
|  | Fever, body pain for 1-2 days | -0.052 (0.044) | 0.95 | 0.87 - 1.03 | 0.235 | -104.897 (88.339) | | -278.038 - 68.243 |
| Risk of severe/serious side effect |  | - | - | - | - | - | | - |
|  | 1 in one crore  1 in one lakh | -0.111 (0.032) | 0.89 | 0.84 - 0.95 | *<0.001* | -223.495 (64.752) | | -350.407 - (-96.583) |
| Vaccine origin | Imported | - | - | - | *-* | - | | - |
|  | Indian product | 0.209 (0.031) | 1.23 | 1.16 - 1.31 | *<0.001* | 419.24 (66.801) | | 288.313 - 550.168 |
| Cost per injection | | -0.0005 (0.00003) | 0.9995 | 0.999 - 0.9996 | *<0.001* | - | | - |

*Supplementary Table 5B: Absence of personal history of COVID-19 infection (n=7518)*

| *Attributes* | |  | *Main Effects Model* | | | *Willingness to Pay (INR)* | |
| --- | --- | --- | --- | --- | --- | --- | --- |
|  |  | *Coefficient (SE)* | *Odds Ratio* | *95% CI* | *P value* | *Coefficient (SE)* | *95% CI* |
| Effectiveness | 50% protection | - | - | - | - | - | - |
|  | 70% protection | 0.645 (0.019) | 1.91 | 1.84 - 1.98 | <0.001 | 954.696 (30.278) | 895.352 - 1014.04 |
|  | 90% protection | 0.977 (0.02) | 2.66 | 2.55 - 2.76 | <0.001 | 1446.319 (36.259) | 1375.252 - 1517.385 |
| Duration of protection | 6 months | - | - | - | - | - | - |
|  | 1 year | 0.316 (0.024) | 1.37 | 1.31 - 1.44 | <0.001 | 467.502 (35.553) | 397.82 -- 537.185 |
|  | 2 years | 0.193 (0.022) | 1.21 | 1.16 - 1.27 | <0.001 | 285.496 (33.034) | 220.751 - 350.242 |
|  | 5 years | 0.362 (0.022) | 1.44 | 1.38 - 1.50 | <0.001 | 535.204 (32.705) | 471.104 - 599.304 |
| Number of Injections | Three Years | - | - | - | - | - | - |
|  | One time | 0.058 (0.019) | 1.06 | 1.02 - 1.10 | 0.002 | 85.829 (27.579) | 31.775 - 139.883 |
|  | Two times | 0.046 (0.019) | 1.05 | 1.01 - 1.09 | 0.015 | 67.667 (27.85) | 13.082 - 122.252 |
| Side effects anticipated | No side effects | - | - | - | - | - | - |
|  | Injection site pain redness and swelling for 1-2 days | -0.128 (0.018) | 0.88 | 0.85 - 0.91 | <0.001 | -188.803 (26.803) | -241.336 - (-136.27) |
|  | Fever, body pain for 1-2 days | -0.076 (0.018) | 0.93 | 0.89 - 0.96 | <0.001 | -112.634 (27.319) | -166.178 - (-59.09) |
| Risk of severe/serious side effect |  | - | - | - | - | -- | - |
|  | 1 in one crore  1 in one lakh | -0.054 (0.013) | 0.95 | 0.92 - 0.97 | <0.001 | -80.182 (19.661) | -118.716 - (-41.648) |
| Vaccine origin |  | - | - | - | - | - | - |
|  | Imported  Indian product | 0.238 (0.013) | 1.27 | 1.24 - 1.30 | <0.001 | 352.463 (20.065) | 313.138 - 391.789 |
| Cost per injection | | -0.001 (0.00001) | 0.999 | 0.999 - 0.999 | <0.001 | - | - |

*Supplementary Table 6a: Severity of previous COVID-19 infection – Asymptomatic (n=418)*

| *Attributes* | |  | | *Main Effects Model* | | | *Willingness to Pay (INR)* | |
| --- | --- | --- | --- | --- | --- | --- | --- | --- |
|  |  | *Coefficient (SE)* | *Odds Ratio* | | *95% CI* | *P value* | *Coefficient (SE)* | *95% CI* |
| Effectiveness | 50% protection | - | - | | - | - | - | - |
|  | 70% protection | 0.649 (0.081) | 1.91 | | 1.63 - 2.24 | *<0.001* | 1014.2 (145.862) | 728.317 - 1300.083 |
|  | 90% protection | 1.047 (0.089) | 2.85 | | 2.39 - 3.39 | *<0.001* | 1635.702 (184.707) | 1273.683 - 1997.722 |
| Duration of protection | 6 months | - | - | | - | *-* | - | - |
|  | 1 year | 0.489 (0.106) | 1.63 | | 1.32 - 2.01 | *<0.001* | 764.039 (170.132) | 430.586 - 1097.493 |
|  | 2 years | 0.422 (0.1) | 1.52 | | 1.25 - 1.85 | *<0.001* | 659.108 (160.534) | 344.467 - 973.749 |
|  | 5 years | 0.472 (0.096) | 1.60 | | 1.33 - 1.94 | *<0.001* | 737.757 (157.67) | 428.729 - 1046.785 |
| Number of Injections | Three times | *-* | *-* | | *-* | *-* | *-* | *-* |
|  | One time | -0.074 (0.084) | 0.93 | | 0.79 - 1.09 | 0.376 | -115.658 (129.566) | -369.603 - 138.288 |
|  | Two times | -0.004 (0.081) | 0.996 | | 0.85 - 1.17 | 0.965 | -5.553 (126.763) | -254.004 - 242.899 |
| Side effects anticipated | No side effects | - | - | | -- | - | - | - |
|  | Injection site pain redness and swelling for 1-2 days | -0.041 (0.08) | 0.96 | | 0.82 - 1.12 | 0.610 | -63.468 (124.269) | -307.031 - 180.095 |
|  | Fever, body pain for 1-2 days | -0.041 (0.082) | 0.96 | | 0.82 - 1.13 | 0.618 | -63.772 (127.895) | -314.442 - 186.898 |
| Risk of severe/serious side effect | 1 in one crore | - | - | | - | - | - | - |
|  | 1 in one lakh | -0.026 (0.058) | 0.97 | | 0.87 - 1.09 | 0.656 | -40.657 (91.152) | -219.312 - 137.997 |
| Vaccine origin | Imported product | - | - | | - | - | - | - |
|  | Indian product | 0.263 (0.058) | 1.30 | | 1.16 - 1.46 | *<0.001* | 411.196 (93.155) | 228.616 - 593.777 |
| Cost per injection | | -0.0006 (0.00006) | 0.999 | | 0.999 - 0.999 | *<0.001* | - | - |

*Supplementary Table 6b: Severity of previous COVID-19 infection – Symptomatic (n=823)*

| *Attributes* | |  | | *Main Effects Model* | | | *Willingness to Pay (INR)* | |
| --- | --- | --- | --- | --- | --- | --- | --- | --- |
|  |  | *Coefficient (SE)* | *Odds Ratio* | | *95% CI* | *P value* | *Coefficient (SE)* | *95% CI* |
| Effectiveness | 50% protection | - | - | | - | - | - | - |
|  | 70% protection | 0.77 (0.053) | 2.16 | | 1.95 - 2.40 | *<0.001* | 1736.922 (170.13) | 1403.474 - 2070.371 |
|  | 90% protection | 1.237 (0.058) | 3.45 | | 3.07 - 3.87 | *<0.001* | 2789.495 (242.152) | 2314.885 - 3264.105 |
| Duration of protection | 6 months | - | - | | - | - | - | - |
|  | 1 year | 0.439 (0.069) | 1.55 | | 1.36 - 1.77 | *<0.001* | 989.722 (165.458) | 665.43 - 1314.013 |
|  | 2 years | 0.422 (0.064) | 1.53 | | 1.35 - 1.73 | *<0.001* | 952.204 (158.714) | 641.131 - 1263.277 |
|  | 5 years | 0.51 (0.062) | 1.66 | | 1.48 - 1.88 | *<0.001* | 1148.691 (160.057) | 834.986 - 1462.397 |
| Number of Injections | Three times | - | - | | - | - | - | - |
|  | One time | 0.023 (0.053) | 1.02 | | 0.92 - 1.14 | 0.658 | 52.8 (119.546) | -181.506 - 287.105 |
|  | Two times | 0.062 (0.055) | 1.06 | | 0.96 - 1.18 | 0.256 | 139.702 (123.986) | -103.306 - 382.711 |
| Side effects anticipated | No side effects | - | - | | - | - | - | - |
|  | Injection site pain redness and swelling for 1-2 days | -0.119 (0.052) | 0.89 | | 0.80 - 0.98 | *0.022* | -267.163 (118.659) | -499.731 – (-34.595) |
|  | Fever, body pain for 1-2 days | -0.059 (0.053) | 0.94 | | 0.85 - 1.05 | 0.262 | -133.336 (118.759) | -366.1 - 99.428 |
| Risk of severe/serious side effect | 1 in one crore | - | - | | - | - | - | - |
|  | 1 in one lakh | -0.147 (0.038) | 0.86 | | 0.80 - 0.93 | *<0.001* | -331.102 (88.913) | -505.368 – (-156.836) |
| Vaccine origin | Imported product | - | - | | - | - | - | - |
|  | Indian product | 0.194 (0.037) | 1.21 | | 1.13 - 1.31 | *<0.001* | 436.241 (91.524) | 256.857 - 615.625 |
| Cost per injection | | -0.0004 (0.00004) | 0.9996 | | 0.999 - 0.9996 | *<0.001* |  | - |

*Supplementary Table 7a: Family COVID-19 history - Yes (n=1259)*

| *Attributes* | |  | | *Main Effects Model* | | | | *Willingness to Pay (INR)* | | |
| --- | --- | --- | --- | --- | --- | --- | --- | --- | --- | --- |
|  |  | *Coefficient (SE)* | *Odds Ratio* | | *95% CI* | *P value* | *Coefficient (SE)* | | *95% CI* |  |
| Effectiveness | 50% protection | - | - | | - | - | - | | - |  |
|  | 70% protection | 0.77 (0.043) | 2.16 | | 1.98 - 2.35 | *<0.001* | 1995.213 (179.422) | | 1643.552 - 2346.874 |  |
|  | 90% protection | 1.221 (0.048) | 3.39 | | 3.09 - 3.72 | *<0.001* | 3163.367 (257.965) | | 2657.765 - 3668.969 |  |
| Duration of protection | 6 months | - | - | | - | - | - | | - |  |
|  | 1 year | 0.433 (0.056) | 1.54 | | 1.38 - 1.72 | *<0.001* | 1121.307 (160.304) | | 807.117 - 1435.497 |  |
|  | 2 years | 0.422 (0.052) | 1.53 | | 1.38 - 1.69 | *<0.001* | 1094.324 (154.063) | | 792.367 - 1396.281 |  |
|  | 5 years | 0.557 (0.051) | 1.75 | | 1.58 - 1.93 | *<0.001* | 1442.669 (164.334) | | 1120.579 - 1764.758 |  |
| Number of Injections | Three times | - | - | | - | - | - | | - |  |
|  | One time | 0.068 (0.043) | 1.07 | | 0.98 - 1.16 | 0.118 | 175.571 (114.198) | | -48.252 - 399.394 |  |
|  | Two times | 0.01 (0.045) | 1.01 | | 0.93 - 1.10 | 0.819 | 26.588 (116.035) | | -200.837 - 254.013 |  |
| Side effects anticipated | No side effects | - | - | | - | - | - | | - |  |
|  | Injection site pain redness and swelling for 1-2 days | -0.079 (0.043) | 0.92 | | 0.85 - 1.00 | 0.064 | -204.199 (111.067) | | -421.886 - 13.488 |  |
|  | Fever, body pain for 1-2 days | -0.083 (0.044) | 0.92 | | 0.84 - 1.00 | 0.057 | -215.258 (113.562) | | -437.836 - 7.319 |  |
| Risk of severe/serious side effect | 1 in one crore | - | - | | - | - | - | | - |  |
|  | 1 in one lakh | -0.115 (0.031) | 0.89 | | 0.84 - 0.95 | *<0.001* | -299.178 (83.67) | | -463.169 – (-135.187) |  |
| Vaccine origin | Imported product | - | - | | - | - | - | | - |  |
|  | Indian product | 0.257 (0.031) | 1.29 | | 1.22 - 1.37 | *<0.001* | 666.909 ( 94.313) | | 482.059 - 851.759 |  |
| Cost per injection | | -0.0004 (0.00003) | 0.9996 | | 0.9996 - 0.9997 | *<0.001* |  | | - |  |

*Supplementary Table 7b: Family COVID-19 history - No (n=7500)*

| *Attributes* | |  | | *Main Effects Model* | | | | *Willingness to Pay (INR)* | | |
| --- | --- | --- | --- | --- | --- | --- | --- | --- | --- | --- |
|  |  | *Coefficient (SE)* | *Odds Ratio* | | *95% CI* | *P value* | *Coefficient (SE)* | | *95% CI* |  |
| Effectiveness | 50% protection | - | - | | - | - | - | | - |  |
|  | 70% protection | 0.641 (0.019) | 1.90 | | 1.83 - 1.97 | *<0.001* | 920.748 (29.203) | | 863.512 - 977.985 |  |
|  | 90% protection | 0.972 (0.02) | 2.64 | | 2.54 - 2.75 | *<0.001* | 1394.825 (34.743) | | 1326.731 - 1462.919 |  |
| Duration of protection | 6 months | - | - | | - | - | - | | - |  |
|  | 1 year | 0.316 (0.024) | 1.37 | | 1.31 - 1.44 | *<0.001* | 453.588 (34.61) | | 385.754 - 521.423 |  |
|  | 2 years | 0.188 (0.022) | 1.21 | | 1.15 - 1.26 | *<0.001* | 269.198 (32.182) | | 206.123 - 332.274 |  |
|  | 5 years | 0.349 (0.022) | 1.42 | | 1.36 - 1.48 | *<0.001* | 501.512 (31.756) | | 439.271 - 563.754 |  |
| Number of Injections | Three times | - | - | | - | - | - | | - |  |
|  | One time | 0.045 (0.019) | 1.05 | | 1.01 - 1.09 | *0.015* | 65.249 (26.876) | | 12.574 - 117.925 |  |
|  | Two times | 0.053 (0.019) | 1.05 | | 1.02 - 1.09 | *0.005* | 76.159 (27.126) | | 22.992 - 129.325 |  |
| Side effects anticipated | No side effects | - | - | | - | - | - | | - |  |
|  | Injection site pain redness and swelling for 1-2 days | -0.132 (0.018) | 0.88 | | 0.85 - 0.91 | *<0.001* | -189.475 (26.152) | | -240.732 – (-138.219) |  |
|  | Fever, body pain for 1-2 days | -0.071 (0.019) | 0.93 | | 0.9 - 0.97 | *<0.001* | -101.585 (26.583) | | -153.687 – (-49.483) |  |
| Risk of severe/serious side effect | 1 in one crore | - | - | | - | - | - | | - |  |
|  | 1 in one lakh | -0.053 (0.013) | 0.95 | | 0.92 - 0.97 | *<0.001* | -76.248 (19.158) | | -113.797 – (-38.7) |  |
| Vaccine origin | Imported product | - | - | | - | - | - | | - |  |
|  | Indian product | 0.231 (0.013) | 1.26 | | 1.23 - 1.29 | *<0.001* | 331.111 (19.437) | | 293.015 - 369.207 |  |
| Cost per injection | | -0.0007 (0.00001) | 0.999 | | 0.999 - 0.999 | *<0.001* |  | | - |  |

*Supplementary Table 8a: Vaccination status - Taken both doses (n=6770)*

| *Attributes* | |  | | *Main Effects Model* | | | | *Willingness to Pay (INR)* | | |
| --- | --- | --- | --- | --- | --- | --- | --- | --- | --- | --- |
|  |  | *Coefficient (SE)* | *Odds Ratio* | | *95% CI* | *P value* | *Coefficient (SE)* | | *95% CI* |  |
| Effectiveness | 50% protection | - | - | | - | - | - | | - |  |
|  | 70% protection | 0.711 (0.02) | 2.04 | | 1.96 - 2.12 | *<0.001* | 1042.742 (32.356) | | 979.326 - 1106.159 |  |
|  | 90% protection | 1.091 (0.022) | 2.98 | | 2.85 - 3.10 | *<0.001* | 1598.618 (39.528) | | 1521.144 - 1676.091 |  |
| Duration of protection | 6 months | - | - | | - | - | - | | - |  |
|  | 1 year | 0.36 (0.025) | 1.43 | | 1.36 - 1.51 | *<0.001* | 528.218 (37.475) | | 454.768 - 601.667 |  |
|  | 2 years | 0.208 (0.023) | 1.23 | | 1.18 - 1.29 | *<0.001* | 305.449 (34.711) | | 237.416 - 373.481 |  |
|  | 5 years | 0.395 (0.023) | 1.48 | | 1.42 - 1.55 | *<0.001* | 579.417 (34.43) | | 511.935 - 646.898 |  |
| Number of Injections | Three times | - | - | | - | - | - | | - |  |
|  | One time | 0.042 (0.02) | 1.04 | | 1.004 - 1.08 | *0.032* | 61.791 (29.021) | | 4.911 - 118.671 |  |
|  | Two times | 0.035 (0.02) | 1.04 | | 0.996 - 1.08 | 0.083 | 50.589 (29.224) | | -6.69 - 107.868 |  |
| Side effects anticipated | No side effects |  |  | |  |  |  | |  |  |
|  | Injection site pain redness and swelling for 1-2 days | -0.134 (0.019) | 0.87 | | 0.84 - 0.91 | *<0.001* | -196.898 (28.037) | | -251.849 – (-141.947) |  |
|  | Fever, body pain for 1-2 days | -0.085 (0.02) | 0.92 | | 0.88 - 0.95 | *<0.001* | -124.642 (28.748) | | -180.987 – (-68.297) |  |
| Risk of severe/serious side effect | 1 in one crore | - | - | | - | - | - | | - |  |
|  | 1 in one lakh | -0.082 (0.014) | 0.92 | | 0.90 - 0.95 | *<0.001* | -119.518 (20.672) | | -160.036 – (-79.001) |  |
| Vaccine origin | Imported product | - | - | | - | - | - | | - |  |
|  | Indian product | 0.260 (0.014) | 1.30 | | 1.26 - 1.33 | *<0.001* | 381.031 (21.188) | | 339.502 - 422.559 |  |
| Cost per injection | | -0.0007 (0.00001) | 0.999 | | 0.999 - 0.999 | *<0.001* |  | | - |  |

*Supplementary Table 8b: Vaccination status - Taken 1^st^ dose (n=1517)*

| *Attributes* | |  | | *Main Effects Model* | | | | *Willingness to Pay (INR)* | | |
| --- | --- | --- | --- | --- | --- | --- | --- | --- | --- | --- |
|  |  | *Coefficient (SE)* | *Odds Ratio* | | *95% CI* | *P value* | *Coefficient (SE)* | | *95% CI* |  |
| Effectiveness | 50% protection | - | - | | - | - | - | | - |  |
|  | 70% protection | 0.52 (0.039) | 1.68 | | 1.56 - 1.82 | *<0.001* | 970.857 (82.552) | | 809.059 - 1132.655 |  |
|  | 90% protection | 0.792 (0.041) | 2.21 | | 2.04 - 2.39 | *<0.001* | 1477.244 (100.489) | | 1280.29 - 1674.198 |  |
| Duration of protection | 6 months | - | - | | - | - | - | | - |  |
|  | 1 year | 0.227 (0.05) | 1.25 | | 1.14 - 1.38 | *<0.001* | 423.516 (94.345) | | 238.604 - 608.428 |  |
|  | 2 years | 0.27 (0.047) | 1.31 | | 1.19 - 1.44 | *<0.001* | 503.425 (90.309) | | 326.423 - 680.427 |  |
|  | 5 years | 0.32 (0.046) | 1.38 | | 1.26 - 1.51 | *<0.001* | 597.073 (88.16) | | 424.283 - 769.863 |  |
| Number of Injections | Three times | - | - | | - | - | - | | - |  |
|  | One time | 0.077 (0.039) | 1.08 | | 1.001 - 1.17 | *0.046* | 143.866 (72.874) | | 1.036 - 286.696 |  |
|  | Two times | 0.082 (0.04) | 1.09 | | 1.004 - 1.17 | *0.039* | 153.8 (74.818) | | 7.158 - 300.442 |  |
| Side effects anticipated | No side effects |  |  | |  |  |  | |  |  |
|  | Injection site pain redness and swelling for 1-2 days | -0.124 (0.039) | 0.88 | | 0.82 - 0.95 | *0.001* | -232.077 (72.884) | | -374.928 – (-89.226) |  |
|  | Fever, body pain for 1-2 days | -0.033 (0.038) | 0.97 | | 0.90 - 1.04 | 0.392 | -61.414 (71.767) | | -202.074 - 79.247 |  |
| Risk of severe/serious side effect | 1 in one crore | - | - | | - | - | - | | - |  |
|  | 1 in one lakh | -0.026 (0.028) | 0.97 | | 0.92 - 1.03 | 0.348 | -49.125 (52.357) | | -151.743 - 53.493 |  |
| Vaccine origin | Imported product | - | - | | - | - | - | | - |  |
|  | Indian product | 0.148 (0.028) | 1.16 | | 1.10 - 1.22 | *<0.001* | 276.562 (53.004) | | 172.676 - 380.447 |  |
| Cost per injection | | -0.0005 (0.00003) | 0.999 | | 0.999 - 0.9995 | *<0.001* |  | | - |  |

*Supplementary Table 8c: Vaccination status - Not taken any dose (n=472)*

| *Attributes* | |  | | *Main Effects Model* | | | | *Willingness to Pay (INR)* | | |
| --- | --- | --- | --- | --- | --- | --- | --- | --- | --- | --- |
|  |  | *Coefficient (SE)* | *Odds Ratio* | | *95% CI* | *P value* | *Coefficient (SE)* | | *95% CI* |  |
| Effectiveness | 50% protection | - | - | | - | - | - | | - |  |
|  | 70% protection | 0.403 (0.076) | 1.50 | | 1.29 - 1.74 | *<0.001* | 599.843 (120.163) | | 364.328 - 835.358 |  |
|  | 90% protection | 0.571 (0.082) | 1.77 | | 1.51 - 2.08 | *<0.001* | 848.454 (135.817) | | 582.258 - 1114.65 |  |
| Duration of protection | 6 months | - | - | | - | - | - | | - |  |
|  | 1 year | 0.41 (0.099) | 1.51 | | 1.24 - 1.83 | *<0.001* | 609.084 (149.393) | | 316.28 - 901.888 |  |
|  | 2 years | 0.287 (0.092) | 1.33 | | 1.11 - 1.59 | *0.002* | 427.003 (138.188) | | 156.159 - 697.846 |  |
|  | 5 years | 0.427 (0.091) | 1.53 | | 1.28 - 1.83 | *<0.001* | 634.985 (139.926) | | 360.734 - 909.235 |  |
| Number of Injections | Three times | - | - | | - | - | - | | - |  |
|  | One time | -0.002 (0.076) | 0.998 | | 0.86 - 1.16 | 0.980 | -2.777 (112.951) | | -224.157 - 218.603 |  |
|  | Two times | 0.039 (0.078) | 1.04 | | 0.89 - 1.21 | 0.620 | 57.563 (116.213) | | -170.21 - 285.336 |  |
| Side effects anticipated | No side effects |  |  | |  |  |  | |  |  |
|  | Injection site pain redness and swelling for 1-2 days | 0.075 (0.075) | 1.08 | | 0.93 - 1.25 | 0.317 | 111.824 (111.828) | | -107.354 - 331.002 |  |
|  | Fever, body pain for 1-2 days | -0.005 (0.078) | 0.99 | | 0.85 - 1.16 | 0.947 | -7.739 (116.093) | | -235.278 - 219.8 |  |
| Risk of severe/serious side effect | 1 in one crore | - | - | | - | - | - | | - |  |
|  | 1 in one lakh | 0.09 (0.055) | 1.09 | | 0.98 - 1.22 | 0.107 | 133.089 (82.588) | | -28.781 - 294.958 |  |
| Vaccine origin | Imported product | - | - | | - | - | - | | - |  |
|  | Indian product | 0.154 (0.054) | 1.17 | | 1.05 - 1.30 | *0.004* | 229.242 (82.197) | | 68.138 - 390.345 |  |
| Cost per injection | | -0.0007 (0.00005) | 0.999 | | 0.999 - 0.999 | *<0.001* |  | | - |  |

*Supplementary Table 9a: SES - Upper class (n=312)*

| *Attributes* | |  | | *Main Effects Model* | | | | *Willingness to Pay (INR)* | | |
| --- | --- | --- | --- | --- | --- | --- | --- | --- | --- | --- |
|  |  | *Coefficient (SE)* | *Odds Ratio* | | *95% CI* | *P value* | *Coefficient (SE)* | | *95% CI* |  |
| Effectiveness | 50% protection | - | - | | - | - | - | | - |  |
|  | 70% protection | 0.482 (0.084) | 1.62 | | 1.37 - 1.91 | *<0.001* | 1251.666 (279.142) | | 704.558 - 1798.774 |  |
|  | 90% protection | 0.821 (0.09) | 2.27 | | 1.91 - 2.71 | *<0.001* | 2130.308 (382.432) | | 1380.755 - 2879.861 |  |
| Duration of protection | 6 months | - | - | | - | - | - | | - |  |
|  | 1 year | 0.157 (0.106) | 1.17 | | 0.95 - 1.44 | 0.141 | 406.66 (280.557) | | -143.222 - 956.542 |  |
|  | 2 years | 0.06 (0.102) | 1.06 | | 0.87 - 1.30 | 0.556 | 156.8 (265.893) | | -364.342 - 677.941 |  |
|  | 5 years | 0.238 (0.1) | 1.27 | | 1.04 - 1.54 | *0.017* | 617.233 (269.479) | | 89.064 - 1145.403 |  |
| Number of Injections | Three times | - | - | | - | - | - | | - |  |
|  | One time | -0.099 (0.085) | 0.91 | | 0.77 - 1.07 | 0.248 | -255.944 (222.981) | | -692.978 - 181.091 |  |
|  | Two times | 0.072 (0.086) | 1.08 | | 0.91 - 1.27 | 0.400 | 188.067 (225.173) | | -253.264 - 629.399 |  |
| Side effects anticipated | No side effects |  |  | |  |  |  | |  |  |
|  | Injection site pain redness and swelling for 1-2 days | -0.05 (0.085) | 0.95 | | 0.81 - 1.12 | 0.559 | -128.913 (220.619) | | -561.318 - 303.493 |  |
|  | Fever, body pain for 1-2 days | 0.076 (0.084) | 1.08 | | 0.91 - 1.27 | 0.366 | 197.786 (221.19) | | -235.739 - 631.31 |  |
| Risk of severe/serious side effect | 1 in one crore | - | - | | - | - | - | | - |  |
|  | 1 in one lakh | -0.092 (0.06) | 0.91 | | 0.81 - 1.03 | 0.127 | -239.707 (159.769) | | -552.849 - 73.436 |  |
| Vaccine origin | Imported product | - | - | | - | - | - | | - |  |
|  | Indian product | 0.167 (0.060) | 1.18 | | 1.05 - 1.33 | *0.005* | 433.263 (166.522) | | 106.885 - 759.64 |  |
| Cost per injection | | -0.0004 (0.00006) | 0.9996 | | 0.9995 - 0.9997 | *<0.001* |  | | - |  |

*Supplementary Table 9b: SES - Middle (n=4703)*

| *Attributes* | |  | | *Main Effects Model* | | | | *Willingness to Pay (INR)* | | |
| --- | --- | --- | --- | --- | --- | --- | --- | --- | --- | --- |
|  |  | *Coefficient (SE)* | *Odds Ratio* | | *95% CI* | *P value* | *Coefficient (SE)* | | *95% CI* |  |
| Effectiveness | 50% protection | - | - | | - | - | - | | - |  |
|  | 70% protection | 0.594 (0.024) | 1.81 | | 1.73 - 1.90 | *<0.001* | 751.161 (31.804) | | 688.826 - 813.495 |  |
|  | 90% protection | 0.878 (0.026) | 2.41 | | 2.29 - 2.53 | *<0.001* | 1109.569 (36.36) | | 1038.304 - 1180.834 |  |
| Duration of protection | 6 months | - | - | | - | - | - | | - |  |
|  | 1 year | 0.278 (0.031) | 1.32 | | 1.24 - 1.40 | *<0.001* | 351.091 (38.899) | | 274.85 - 427.331 |  |
|  | 2 years | 0.13 (0.029) | 1.14 | | 1.08 - 1.20 | *<0.001* | 163.666 (36.266) | | 92.586 - 234.745 |  |
|  | 5 years | 0.324 (0.028) | 1.38 | | 1.31 - 1.46 | *<0.001* | 409.958 (35.751) | | 339.888 - 480.029 |  |
| Number of Injections | Three times | - | - | | - | - | - | | - |  |
|  | One time | 0.023 (0.024) | 1.02 | | 0.98 - 1.07 | 0.345 | 28.683 (30.431) | | -30.96 - 88.325 |  |
|  | Two times | 0.048 (0.024) | 1.05 | | 1.0004 - 1.10 | *0.048* | 60.448 (30.641) | | 0.393 - 120.504 |  |
| Side effects anticipated | No side effects |  |  | |  |  |  | |  |  |
|  | Injection site pain redness and swelling for 1-2 days | -0.137 (0.023) | 0.87 | | 0.83 - 0.91 | *<0.001* | -173.33 (29.624) | | -231.391 – (-115.268) |  |
|  | Fever, body pain for 1-2 days | -0.097 (0.024) | 0.91 | | 0.87 - 0.95 | *<0.001* | -122.253 (30.108) | | -181.265 – (-63.242) |  |
| Risk of severe/serious side effect | 1 in one crore | - | - | | - | - | - | | - |  |
|  | 1 in one lakh | -0.003 (0.017) | 0.997 | | 0.96 - 1.03 | 0.883 | -3.185 (21.659) | | -45.636 - 39.267 |  |
| Vaccine origin | Imported product | - | - | | - | - | - | | - |  |
|  | Indian product | 0.206 (0.017) | 1.23 | | 1.19 - 1.27 | *<0.001* | 260.813 (21.594) | | 218.49 - 303.137 |  |
| Cost per injection | | -0.0008 (0.00002) | 0.999 | | 0.999 - 0.999 | *<0.001* |  | | - |  |

*Supplementary Table 9c: SES - Lower (n=3744)*

| *Attributes* | |  | | *Main Effects Model* | | | | *Willingness to Pay (INR)* | | |
| --- | --- | --- | --- | --- | --- | --- | --- | --- | --- | --- |
|  |  | *Coefficient (SE)* | *Odds Ratio* | | *95% CI* | *P value* | *Coefficient (SE)* | | *95% CI* |  |
| Effectiveness | 50% protection | - | - | | - | - | - | | - |  |
|  | 70% protection | 0.76 (0.026) | 2.14 | | 2.03 - 2.25 | *<0.001* | 1490.575 (66.109) | | 1361.003 - 1620.147 |  |
|  | 90% protection | 1.19 (0.028) | 3.29 | | 3.11 - 3.47 | *<0.001* | 2332.408 (88.969) | | 2158.033 - 2506.783 |  |
| Duration of protection | 6 months | - | - | | - | - | - | | - |  |
|  | 1 year | 0.417 (0.033) | 1.52 | | 1.42 - 1.62 | *<0.001* | 817.309 (68.081) | | 683.873 - 950.746 |  |
|  | 2 years | 0.354 (0.031) | 1.42 | | 1.34 - 1.51 | *<0.001* | 693.491 (63.585) | | 568.866 - 818.116 |  |
|  | 5 years | 0.462 (0.03) | 1.59 | | 1.50 - 1.68 | *<0.001* | 906.21 (63.724) | | 781.313 - 1031.107 |  |
| Number of Injections | Three times | - | - | | - | - | - | | - |  |
|  | One time | 0.094 (0.026) | 1.10 | | 1.04 - 1.15 | *<0.001* | 184.126 (51.098) | | 83.976 - 284.276 |  |
|  | Two times | 0.047 (0.026) | 1.05 | | 0.995 - 1.10 | 0.075 | 91.628 (51.778) | | -9.855 - 193.112 |  |
| Side effects anticipated | No side effects |  |  | |  |  |  | |  |  |
|  | Injection site pain redness and swelling for 1-2 days | -0.116 (0.025) | 0.89 | | 0.85 - 0.94 | *<0.001* | -227.726 (49.522) | | -324.788 – (-130.665) |  |
|  | Fever, body pain for 1-2 days | -0.061 (0.026) | 0.94 | | 0.89 - 0.99 | *0.017* | -119.572 (50.308) | | -218.175 – (-20.97) |  |
| Risk of severe/serious side effect | 1 in one crore | - | - | | - | - | - | | - |  |
|  | 1 in one lakh | -0.134 (0.019) | 0.87 | | 0.84 - 0.91 | *<0.001* | -262.919 (37.111) | | -335.656 – (-190.182) |  |
| Vaccine origin | Imported product | - | - | | - | - | - | | - |  |
|  | Indian product | 0.280 (0.018) | 1.32 | | 1.28 - 1.37 | *<0.001* | 548.694 (39.871) | | 470.548 - 626.84 |  |
| Cost per injection | | -0.0005 (0.00002) | 0.999 | | 0.999 - 0.9995 | *<0.001* |  | | - |  |

*Supplementary Table 10a: Healthcare worker - Yes (n=1296)*

| *Attributes* | |  | | *Main Effects Model* | | | | *Willingness to Pay (INR)* | | |
| --- | --- | --- | --- | --- | --- | --- | --- | --- | --- | --- |
|  |  | *Coefficient (SE)* | *Odds Ratio* | | *95% CI* | *P value* | *Coefficient (SE)* | | *95% CI* |  |
| Effectiveness | 50% protection | - | - | | - | - | - | | - |  |
|  | 70% protection | 0.832 (0.043) | 2.30 | | 2.11 - 2.50 | *<0.001* | 2302.356 (212.787) | | 1885.301 - 2719.412 |  |
|  | 90% protection | 1.35 (0.048) | 3.86 | | 3.51 - 4.24 | *<0.001* | 3736.308 (321.077) | | 3107.009 - 4365.608 |  |
| Duration of protection | 6 months | - | - | | - | - | - | | - |  |
|  | 1 year | 0.33 (0.055) | 1.39 | | 1.25 - 1.55 | *<0.001* | 913.916 (162.073) | | 596.259 - 1231.572 |  |
|  | 2 years | 0.377 (0.052) | 1.46 | | 1.32 - 1.62 | *<0.001* | 1044.474 (162.395) | | 726.185 - 1362.763 |  |
|  | 5 years | 0.601 (0.05) | 1.82 | | 1.65 - 2.01 | *<0.001* | 1663.533 (186.853) | | 1297.308 - 2029.758 |  |
| Number of Injections | Three times | - | - | | - | - | - | | - |  |
|  | One time | 0.074 (0.043) | 1.08 | | 0.99 - 1.17 | 0.087 | 205.518 (122.362) | | -34.306 - 445.342 |  |
|  | Two times | 0.131 (0.045) | 1.14 | | 1.04 - 1.24 | *0.003* | 362.678 (128.4) | | 111.019 - 614.336 |  |
| Side effects anticipated | No side effects |  |  | |  |  |  | |  |  |
|  | Injection site pain redness and swelling for 1-2 days | -0.11 (0.042) | 0.90 | | 0.82 - 0.97 | *0.009* | -304.764 (119.533) | | -539.044 – (-70.483) |  |
|  | Fever, body pain for 1-2 days | 0.042 (0.043) | 1.04 | | 0.96 - 1.13 | 0.328 | 116.282 (119.054) | | -117.06 - 349.624 |  |
| Risk of severe/serious side effect | 1 in one crore | - | - | | - | - | - | | - |  |
|  | 1 in one lakh | -0.134 (0.031) | 0.87 | | 0.82 - 0.93 | *<0.001* | -370.081 (91.361) | | -549.145 – (-191.017) |  |
| Vaccine origin | Imported product | - | - | | - | - | - | | - |  |
|  | Indian product | 0.280 (0.031) | 1.32 | | 1.25 - 1.41 | *<0.001* | 775.374 (105.813) | | 567.985 - 982.763 |  |
| Cost per injection | | -0.0004 (0.00003) | 0.9996 | | 0.9996 - 0.9997 | *<0.001* |  | | - |  |

*Supplementary Table 10b: Healthcare worker - No (n=7463)*

| *Attributes* | |  | | *Main Effects Model* | | | | *Willingness to Pay (INR)* | | |
| --- | --- | --- | --- | --- | --- | --- | --- | --- | --- | --- |
|  |  | *Coefficient (SE)* | *Odds Ratio* | | *95% CI* | *P value* | *Coefficient (SE)* | | *95% CI* |  |
| Effectiveness | 50% protection | - | - | | - | - | - | | - |  |
|  | 70% protection | 0.632 (0.019) | 1.88 | | 1.81 - 1.95 | *<0.001* | 901.611 (28.99) | | 844.791 - 958.432 |  |
|  | 90% protection | 0.95 (0.02) | 2.59 | | 2.49 - 2.69 | *<0.001* | 1356.581 (34.227) | | 1289.497 - 1423.665 |  |
| Duration of protection | 6 months | - | - | | - | - | - | | - |  |
|  | 1 year | 0.333 (0.024) | 1.39 | | 1.33 - 1.46 | *<0.001* | 474.682 (34.633) | | 406.802 - 542.562 |  |
|  | 2 years | 0.196 (0.022) | 1.22 | | 1.16 - 1.27 | *<0.001* | 279.851 (32.093) | | 216.951 - 342.752 |  |
|  | 5 years | 0.342 (0.022) | 1.41 | | 1.35 - 1.47 | *<0.001* | 488.506 (31.682) | | 426.411 - 550.602 |  |
| Number of Injections | Three times | - | - | | - | - | - | | - |  |
|  | One time | 0.045 (0.019) | 1.05 | | 1.01 - 1.08 | *0.017* | 63.611 (26.77) | | 11.143 - 116.08 |  |
|  | Two times | 0.032 (0.019) | 1.03 | | 0.99 - 1.07 | 0.091 | 45.541 (27.021) | | -7.419 - 98.501 |  |
| Side effects anticipated | No side effects |  |  | |  |  |  | |  |  |
|  | Injection site pain redness and swelling for 1-2 days | -0.129 (0.018) | 0.88 | | 0.85 - 0.91 | *<0.001* | -184.142 (26.057) | | -235.214 - -133.071 |  |
|  | Fever, body pain for 1-2 days | -0.099 (0.019) | 0.91 | | 0.87 - 0.94 | *<0.001* | -141.164 (26.574) | | -193.248 - -89.081 |  |
| Risk of severe/serious side effect | 1 in one crore | - | - | | - | - | - | | - |  |
|  | 1 in one lakh | -0.052 (0.013) | 0.95 | | 0.93 - 0.97 | *<0.001* | -73.645 (19.112) | | -111.104 - -36.186 |  |
| Vaccine origin | Imported product | - | - | | - | - | - | | - |  |
|  | Indian product | 0.229 (0.013) | 1.26 | | 1.23 - 1.29 | *<0.001* | 326.875 (19.34) | | 288.97 - 364.781 |  |
| Cost per injection | | -0.0007 (0.00001) | 0.999 | | 0.999 - 0.999 | *<0.001* |  | | - |  |

*Supplementary Table 11: Sensitivity Analyses based on educated guesses**

| *Attributes* | | *Main Effects Model* | | | *Willingness to Pay (INR)* | |  |
| --- | --- | --- | --- | --- | --- | --- | --- |
|  |  | *Odds Ratio* | *95% CI* | *P value* | *Coefficient (SE)* | *95% CI* |  |
| Effectiveness | 50% protection | - | - | - | - | - |  |
|  | 70% protection | 2.11 | 1.98 - 2.24 | **<0.001** | 1774.353 (110.731) | 1557.325 - 1991.381 |  |
|  | 90% protection | 3.26 | 3.04 - 3.48 | **<0.001** | 2804.921 (155.67) | 2499.813 - 3110.029 |  |
| Duration of protection | 6 months | - | - | - | - | - |  |
|  | 1 year | 1.48 | 1.37 - 1.60 | **<0.001** | 930.276 (103.22) | 727.969 - 1132.582 |  |
|  | 2 years | 1.47 | 1.36 - 1.58 | **<0.001** | 912.671 (99.322) | 718.004 - 1107.337 |  |
|  | 5 years | 1.67 | 1.55 - 1.79 | **<0.001** | 1213.19 (103.279) | 1010.767 - 1415.613 |  |
| Number of Injections | Three times | - | - | - | - | - |  |
|  | One time | 1.05 | 0.98 - 1.11 | 0.161 | 105.205 (75.778) | -43.317 - 253.727 |  |
|  | Two times | 1.05 | 0.98 - 1.12 | 0.167 | 107.051 (77.833) | -45.499 - 259.601 |  |
| Side effects anticipated | No side effects |  |  |  |  |  |  |
|  | Injection site pain redness and swelling for 1-2 days | 0.90 | 0.85 - 0.96 | **0.001** | -246.438 (74.703) | -392.854 – (-100.022) |  |
|  | Fever, body pain for 1-2 days | 0.95 | 0.89 - 1.01 | 0.107 | -120.638 (75.022) | -267.679 - 26.403 |  |
| Risk of severe/serious side effect | 1 in one crore | - | - | - | - | - |  |
|  | 1 in one lakh | 0.89 | 0.85 - 0.93 | **<0.001** | -288.594 (55.78) | -397.921 – (-179.266) |  |
| Vaccine origin | Imported product | - | - | - | - | - |  |
|  | Indian product | 1.28 | 1.22 - 1.33 | **<0.001** | 578.048 (60.716) | 459.047 - 697.05 |  |
| Cost per injection | | 0.9996 | 0.9995 - 0.9996 | **<0.001** | - | - |  |

* Defined as those who were healthcare workers involved in patient interaction, those with past history of symptomatic COVID-19 or family history of COVID-19

*Supplementary Table 12: Sensitivity Analyses including all respondents (N = 10,000)*

| **Attributes (N=10000)** | | **Main Effects Model** | | | | **Willingness to Pay (INR)** | |
| --- | --- | --- | --- | --- | --- | --- | --- |
|  |  | **Coefficient (SE)** | **Odds Ratio** | **95% CI** | ***P* value** | **Coefficient (SE)** | **95% CI** |
| **Effectiveness** | 50% protection | **-** | **-** | **-** | **-** | **-** | **-** |
|  | 70% protection | 0.42(0.01) | 1.52 | 1.48-1.56 | <0.001 | 1109.56(41.79) | 1027.66-1191.47 |
|  | 90% protection | 0.6(0.01) | 1.83 | 1.78-1.88 | <0.001 | 1603.12(46.43) | 1512.12-1694.12 |
| **Duration of protection** | 6 months | **-** | **-** | **-** | **-** | **-** | **-** |
|  | 1 year | 0.2(0.02) | 1.22 | 1.19-1.26 | <0.001 | 531.22(40.88) | 451.1-611.35 |
|  | 2 years | 0.15(0.01) | 1.16 | 1.13-1.2 | <0.001 | 405.46(38.72) | 329.58-481.34 |
|  | 5 years | 0.27(0.01) | 1.31 | 1.27-1.35 | <0.001 | 721.5(41.45) | 640.27-802.73 |
| **Number of Injections** | Three times | **-** | **-** | **-** | **-** | **-** | **-** |
|  | One time | 0.12(0.01) | 1.13 | 1.1-1.16 | <0.001 | 318.56(34.27) | 251.4-385.72 |
|  | Two times | 0.05(0.01) | 1.05 | 1.02-1.07 | <0.001 | 122.86(34.48) | 55.27-190.44 |
| **Vaccine origin** | Imported | **-** | **-** | **-** | **-** | **-** | **-** |
|  | Indian | 0.19(0.01) | 1.21 | 1.19-1.24 | <0.001 | 512.25(28.49) | 456.41-568.08 |
| **Common side effects anticipated** | No side effects | **-** | **-** | **-** | **-** | **-** | **-** |
|  | Injection site pain redness and swelling for 1-2 days | -0.14(0.01) | 0.87 | 0.84-0.89 | <0.001 | -383.3(34.74) | -451.38--315.21 |
|  | Fever, body pain for 1-2 days | -0.1(0.01) | 0.90 | 0.88-0.92 | <0.001 | -278.9(33.96) | -345.46--212.34 |
| **Risk of severe/serious side effect** | One in one crore (10 million) | **-** | **-** | **-** | **-** | **-** | **-** |
|  | One in one lakh (100 thousand) | -0.05(0.01) | 0.95 | 0.93-0.97 | <0.001 | -145.31(27.62) | -199.44--91.17 |
| **Cost per injection** | | -0.00038(0.00001) | 1.00 | 1-1 | <0.001 | **-** | **-** |

*Supplementary Table 13: Sensitivity analysis restricted to respondents who failed the trap question (N = 1,241)*

| **Attributes (N=1241)** | | **Main Effects Model** | | | | **Willingness to Pay (INR)** | |
| --- | --- | --- | --- | --- | --- | --- | --- |
|  |  | **Coefficient (SE)** | **Odds Ratio** | **95% CI** | ***P* value** | **Coefficient (SE)** | **95% CI** |
| **Effectiveness** | 50% protection | **-** | **-** | **-** | **-** | **-** | **-** |
|  | 70% protection | -0.03(0.03) | 0.97 | 0.91-1.03 | 0.339 | 2394.85(4213.85) | -5864.15-10653.84 |
|  | 90% protection | 0.07(0.03) | 1.07 | 1-1.14 | 0.052 | -4892.81(7792.57) | -20165.97-10380.36 |
| **Duration of protection** | 6 months | **-** | **-** | **-** | **-** | **-** | **-** |
|  | 1 year | 0.05(0.04) | 1.05 | 0.97-1.14 | 0.209 | -3715.07(6229.41) | -15924.49-8494.35 |
|  | 2 years | 0.05(0.04) | 1.05 | 0.98-1.13 | 0.187 | -3777.85(6388.56) | -16299.19-8743.5 |
|  | 5 years | 0.16(0.04) | 1.17 | 1.09-1.26 | 0.000 | -11712.54(17480.02) | -45972.74-22547.67 |
| **Number of Injections** | Three times | **-** | **-** | **-** | **-** | **-** | **-** |
|  | One time | 0.04(0.03) | 1.05 | 0.98-1.11 | 0.178 | -3285.06(5212.52) | -13501.42-6931.29 |
|  | Two times | -0.14(0.03) | 0.87 | 0.81-0.93 | <0.001 | 10395.16(15486.98) | -19958.77-40749.08 |
| **Vaccine origin** | Imported | **-** | **-** | **-** | **-** | **-** | **-** |
|  | Indian | -0.15(0.03) | 0.86 | 0.8-0.92 | <0.001 | 11345.13(16667.11) | -21321.81-44012.07 |
| **Common side effects anticipated** | No side effects | **-** | **-** | **-** | **-** | **-** | **-** |
|  | Injection site pain redness and swelling for 1-2 days | -0.12(0.03) | 0.89 | 0.83-0.95 | 0.001 | 8569.43(12700.33) | -16322.76-33461.62 |
|  | Fever, body pain for 1-2 days | -0.07(0.03) | 0.93 | 0.88-0.98 | 0.008 | 5465.52(8243.72) | -10691.87-21622.92 |
| **Risk of severe/serious side effect** | One in one crore (10 million) | **-** | **-** | **-** | **-** | **-** | **-** |
|  | One in one lakh (100 thousand) | 0.19(0.03) | 1.21 | 1.14-1.27 | <0.001 | -13897.66(20389.09) | -53859.55-26064.23 |
| **Cost per injection** | | 0.000013(0.00002) | 1.00 | 1-1 | 0.492 | **-** | **-** |
